# Palbociclib releases the latent differentiation capacity of neuroblastoma cells

**DOI:** 10.1016/j.devcel.2023.08.028

**Published:** 2023-09-20

**Authors:** Kirsty M. Ferguson, Sarah L. Gillen, Lewis Chaytor, Evon Poon, Daniel Marcos, Roshna Lawrence Gomez, Laura M. Woods, Lidiya Mykhaylechko, Louis Elfari, Barbara Martins da Costa, Yann Jamin, Jason S. Carroll, Louis Chesler, Fahad R. Ali, Anna Philpott

**Affiliations:** 1 Wellcome-MRC Cambridge Stem Cell Institute, Jeffrey Cheah Biomedical Centre, Cambridge Biomedical Campus, Cambridge CB2 0AW, UK; 2 Department of Oncology, University of Cambridge, Cambridge CB2 0XZ, UK; 3 Division of Clinical Studies, The Institute of Cancer Research (ICR) and Royal Marsden NHS Trust, Sutton SM2 5NG, UK; 4 College of Medicine, Mohammed Bin Rashid University of Medicine and Health Sciences, Dubai Healthcare City, P.O. Box 505055, Dubai, United Arab Emirates; 5 Wellcome-MRC Cambridge Stem Cell Institute Advanced Imaging Facility, Cambridge CB2 0AW, UK; 6 Division of Radiotherapy and Imaging, The Institute of Cancer Research (ICR) and Royal Marsden NHS Trust, Sutton SM2 5NG, UK; 7 Cancer Research UK Cambridge Institute, University of Cambridge, Cambridge CB2 0RE, UK

## Abstract

Neuroblastoma is the most common extracranial solid tumor in infants, arising from developmentally stalled neural crest-derived cells. Driving tumor differentiation is a promising therapeutic approach for this devastating disease. Here, we show that the CDK4/6 inhibitor palbociclib not only inhibits proliferation but induces extensive neuronal differentiation of adrenergic neuroblastoma cells. Palbociclib-mediated differentiation is manifested by extensive phenotypic and transcriptional changes accompanied by the establishment of an epigenetic program driving expression of mature neuronal features. *In vivo* palbociclib significantly inhibits tumor growth in mouse neuroblastoma models. Furthermore, dual treatment with retinoic acid resets the oncogenic adrenergic core regulatory circuit of neuroblastoma cells, further suppresses proliferation, and can enhance differentiation, altering gene expression in ways that significantly correlate with improved patient survival. We therefore identify palbociclib as a therapeutic approach to dramatically enhance neuro-blastoma differentiation efficacy that could be used in combination with retinoic acid to improve patient outcomes.

## Graphical abstract

**Graphical abstract:**
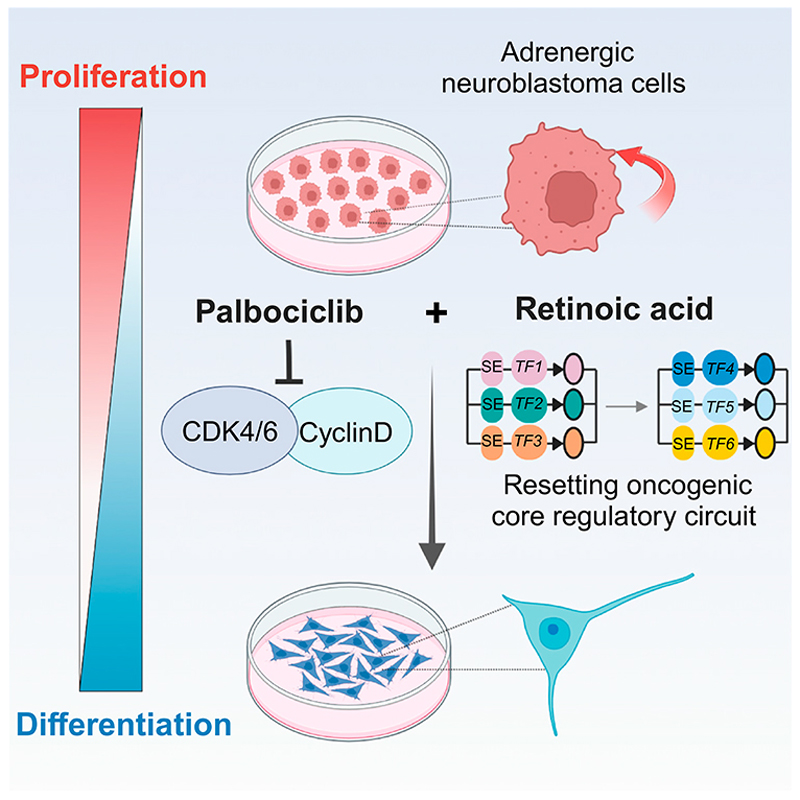

## Introduction

Neuroblastoma is the most common extracranial solid tumor in infants, accounting for 15% of pediatric cancer deaths. In normal development, sympathoadrenal precursor cells derived from the neural crest differentiate into cell types including sympathetic neurons, adrenal chromaffin cells, and Schwann cells. In neuroblastoma, these sympathetic precursor cells fail to differentiate and are locked into an immature state that drives tumor growth.^1,2^ However, a subset of tumors can undergo spontaneous remission linked to tumor cell differentiation.^3–5^ Neuroblastoma therefore presents a unique opportunity whereby differentiation therapies that reactivate normal developmental processes provide a promising therapeutic approach.^2^ The promise of differentiation therapies is exemplified by pediatric acute pro-myelocytic leukemia where introduction of all-*trans* retinoic acid (herein referred to as RA, an active metabolite of 13-*cis* RA), a known differentiation-inducing agent, into treatment regimens has dramatically improved complete remission rates.^6,7^ While retinoid treatments (namely 13-*cis* RA) are currently part of the treatment regimen for high-risk neuroblastoma, this treatment has varied efficacy in patients and is limited to maintenance therapy for minimal residual disease, following aggressive chemo/radiotherapy.^8–11^ Novel, kinder therapies are therefore needed to treat this devastating childhood cancer, and reactivating a latent ability to undergo differentiation is a promising potential approach.^12,13^

While neuroblastomas have a low mutational burden, they have a strong epigenetic component; their pro-proliferative state and lineage identity is driven by a core regulatory circuitry (CRC) of transcription factors, themselves driven by clusters of enhancers often referred to as “super-enhancers” (SEs).^14,15^ Neuroblastomas have been found to contain two cell types, namely adrenergic (ADRN) and mesenchymal (MES), which reflect different developmental stages of the sympathoadrenergic lineage,^15^ and where each cell type is maintained by a distinct CRC and set of SEs. While ADRN- and MES-type cells can spontaneously interconvert,^16^ ADRN cells are the most tumorigenic.^15,17^ ADRN tumors, encompassing both MYCN-amplified and non-amplified diseases, are stalled in a noradrenergic sympathetic neuronal precursor state maintained by CRC transcription factors including ASCL1, PHOX2B, GATA3, and HAND2. If the correct cues can be identified, these tumors may be driven to re-enter a post-mitotic differentiated state.

Therapeutically tipping the balance from proliferation to differentiation is likely to require the following: (1) driving cell-cycle exit, via downregulation of cell-cycle genes and up-regulation of CDK (cyclin-dependent kinase) inhibitors, (2) driving upregulation of differentiation genes, and (3) resetting of the CRC gene network in favor of a stable differentiated state. Many ADRN neuroblastoma cell lines have shown neuronal differentiation *in vitro* in response to treatment with all-*trans* RA,^18,19^ with RA recently shown to reset the CRC network in NMyc-driven neuroblastoma cells.^20,21^ Previous studies have also indicated that lengthening of the cell cycle in neuronal precursors promotes differentiation.^22–24^ In addition, several preclinical studies suggest CDK inhibitors as promising treatments in neuroblastoma on the basis of their anti-proliferative functions.^6,25–28^ While clinical application of first generation pan-CDK inhibitors has generally proven unsuccessful in cancer therapeutic applications due to low specificity and off-target effects, several specific CDK inhibitors have now been developed.^29^ Palbociclib (PB), a CDK4/6 inhibitor (Ibrance, Pfizer; PD-0332991) approved for front-line treatment of HR-positive and HER2-negative breast cancers in combination with endocrine therapy,^30^ has previously been shown to induce G1 arrest in neuroblastoma.^26,27,31^ Extensive overexpression of cyclin D-CDK4/6 components in neuroblastoma makes this a particularly attractive therapeutic target.^32^

Here, we investigate PB as a clinically relevant therapy for neuroblastoma. We find that PB resets the global transcriptional and epigenetic landscapes of ADRN-type neuroblastoma cells, resulting in a dual phenotypic endpoint that strongly favors neuronal differentiation while simultaneously decreasing proliferation. We confirm PB is an effective agent resulting in significant survival benefit *in vivo* in mice. Additional features of an enduring post-mitotic differentiated state can be achieved in neuroblastoma cells by combining PB and RA, which act to significantly reset the oncogenic CRC in favor of differentiation. We therefore propose that PB, alone or as a combinatorial therapy with RA, could significantly improve neuroblastoma patient outcomes by re-engaging an anti-tumorigenic differentiation program.

## Results

### PB inhibits proliferation and promotes differentiation in ADRN neuroblastoma cells

**Figure 1:**
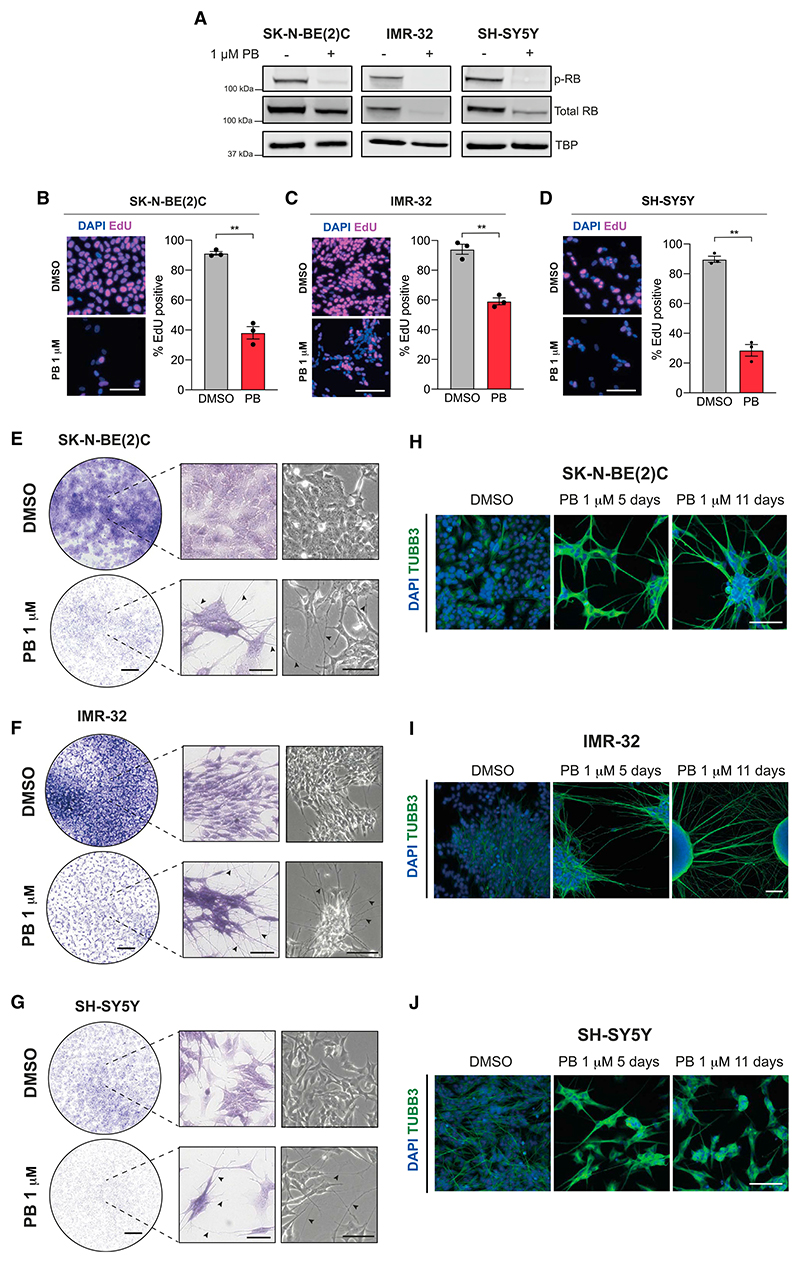
Palbociclib drives cell-cycle exit and differentiation in adrenergic neuroblastoma cells (A) Western blot analysis of phospho-RB and total RB protein levels in SK-N-BE(2)C, IMR-32, andSH-SY5Y cells, untreated and treated with 1 μM palbociclib for 24 h. TBP was used as a housekeeping loading control. (B–D) Representative fluorescent images of EdU incorporation following a 24-h pulse in (B) SK-N-BE(2)C, (C) IMR-32, and (D) SH-SY5Y cells (pulse began day 4 of 5-day treatment with vehicle [DMSO] or palbociclib [1 μM]). Scale bars: 100 μm. Analysis of % cells with EdU incorporation. n = 3 biological replicates, mean ± SEM. **p ≤ 0.01, one-tailed paired t test. DAPI nuclear counterstain (blue). (E–G) Crystal violet staining of (E) SK-N-BE(2)C, (F) IMR-32, and (G) SH-SY5Y cells treated with palbociclib or DMSO vehicle control for 5 days. Representative of n = 3 biological replicates. Right-hand images show representative phase-contrast images prior to fixation. Arrows indicate examples of neurite extension. Scale bars: 2 mm and 100 μm. (H–J) Immunocytochemistry analysis of TUBB3 (green) expression in (H) SK-N-BE(2)C, (I) IMR-32, and (J) SH-SY5Y and cells following 5 or 11 days of palbociclib treatment. Scale bars: 100 μm. DAPI nuclear counterstain (blue). See also Figure S1 and Videos S1, S2, and S3.

Given the known links between cell-cycle regulation in division and differentiation in the developing nervous system and in neuroblastoma,^23,32–34^ we first investigated the effects of the CDK4/6 inhibitor PB on ADRN-type neuroblastoma cell lines SK-N-BE(2)C, IMR-32, and SH-SY5Y. Our aim is to find a therapy that will reactivate the latent differentiation potential of neuroblastoma cells in a variety of genetic backgrounds. We therefore chose cell lines that cover a mixture of back-grounds that are all CDK4/6 wild type (WT)^28^ with a range of CCND1 expression levels^26^: SK-N-BE(2)C—1p loss, TP53 mutant, MYCN amplified^35^; IMR-32—MYCN amplified, MEIS1 amplified, 1p loss^36,37^; and SH-SY5Y—ALK mutant F1174L, KRAS(G12V),^38^ 1q segment gain, 17q gain.^36,39^ Cells were treated with 1 μM PB, a standard dosage used in cellular studies^40,41^ that is similar to the IC50s of all three lines (Figure S1A). The cyclin D-CDK4/6 complex phosphorylates RB,^42,43^ a tumor suppressor that blocks the G1 to S phase transition, so reduced RB phosphorylation is an indicator of CDK4/6 inhibition. As expected, in all cell lines, 24-h PB treatment resulted in hypo-phosphorylation of RB (Figure 1A). We also see a reduction in the total RB level, as has been observed previously with CDK4/6 knockdown in neuroblastoma cell lines.^44^ Consistently, 5 days of PB treatment resulted in a significant decrease in proliferation, compared with the vehicle control, as shown by EdU incorporation and crystal violet staining (Figures 1B–1G). Interestingly, we also observed coincident neuronal differentiation features upon PB treatment, namely neurite outgrowth and cell clustering (Figures 1E–1G). Live-cell imaging shows a direct change in morphology during PB treatment, with negligible cell death (Videos S1, S2, and S3). Automated segmentation of neurite outgrowth using live-cell NeuroTrack analysis confirmed an increase in neurite length and branchpoints with PB treatment compared with the DMSO control in all cell lines (Figure S1B). In contrast, the MES-type SH-EP and GIMEN neuroblastoma cell lines displayed decreased proliferation but no features of neuronal differentiation upon PB treatment (Figures S1C–S1F), suggesting that the pro-differentiation effect is limited to ADRN-type neuroblastoma models. Immunocytochemistry analysis of SK-N-BE(2)C, IMR-32, and SH-SY5Y cells following 5 or 11 days PB treatment confirmed neurite outgrowth accompanied by up-regulation of the classical neuronal marker βIII-tubulin (TUBB3) (Figures 1H and 1J). These data demonstrate that PB not only drives cell-cycle exit, as expected, but also induces neuronal differentiation of ADRN-type neuroblastoma cells.

### PB activates a transcriptional program of neuronal differentiation

**Figure 2:**
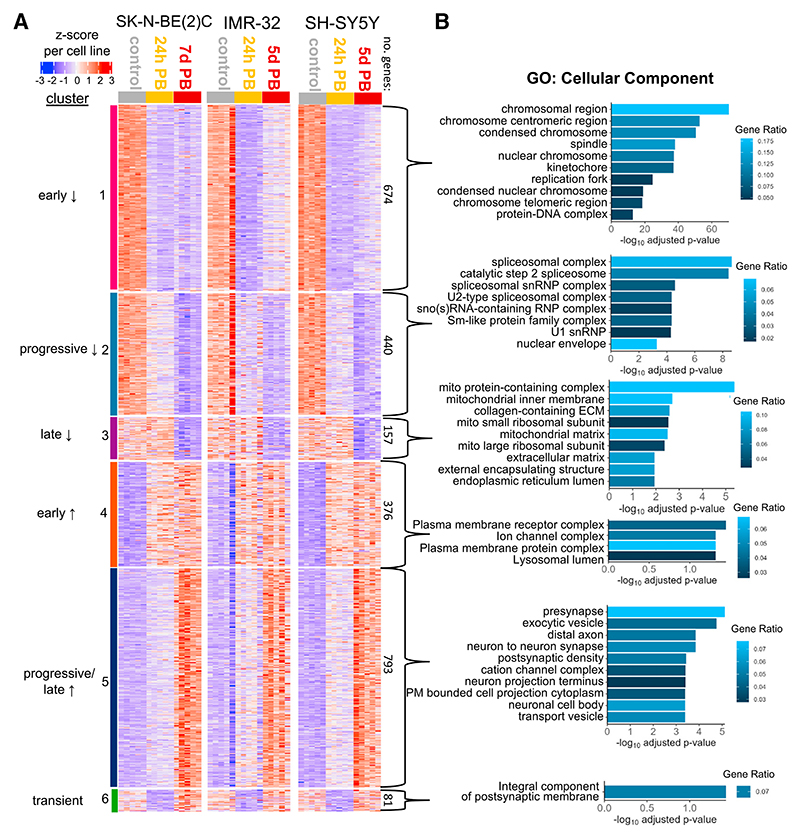
Palbociclib drives transcriptional changes associated with differentiation of neuroblastoma (A) Heatmap of all genes differentially expressed across the three cell lines for five biological replicates of control, 24-h PB, and 5/7 days PB samples. The *Z* score scaling for each gene has been applied internally per cell line. Clusters were assigned using k-means clustering on the scaled data from all cell lines. The number of genes in each cluster is indicated. (B) Gene ontology analysis for cellular components using clusterProfiler, conducted for each cluster of genes in (A). Terms are ordered by the adjusted p value; the 10 most significant terms are shown. See also Figure S2.

We next sought to characterize how PB alters the transcriptional landscape of neuroblastoma cells. SK-N-BE(2)C, IMR-32, and SH-SY5Y cell lines were treated with PB for short (24 h) or longer (5–7 days) periods and RNA collected for RNA sequencing (RNA-seq) (Figure S2A). PB efficacy was confirmed by a strong down-regulation of E2F target genes (directly regulated by phospho-RB) at the 24-h time point (Figure S2B), providing support for the anti-proliferative effects of PB. For each cell line, differential expression analysis was conducted using DESeq2 to identify rapidly and more slowly responding genes.^45^ Clustering of these genes after normalization within each cell line identified six sets of genes that differed in response to PB; importantly, the PB response of each gene cluster is similar across cell lines (Figure 2A). Gene ontology analysis on genes within these clusters showed early downregulation of cell-cycle-related genes and progressive downregulation of the spliceosome with later down-regulation of mitochondrial components (Figures 2A and 2B); this is consistent with the decreased proliferation we observed and perhaps reflective of changes in mitochondrial dynamics known to occur as cells differentiate.^46^ Early upregulation of plasma membrane and ion channel complexes was also observed, followed later by upregulation of synapse and axon components indicative of differentiation (Figures 2A and 2B). Together these data show a robust reprogramming of the transcriptome by PB toward reduced proliferation and induction of neuronal differentiation in all three neuroblastoma cell lines.

### PB rewires the epigenetic landscape consistent with differentiation

**Figure 3:**
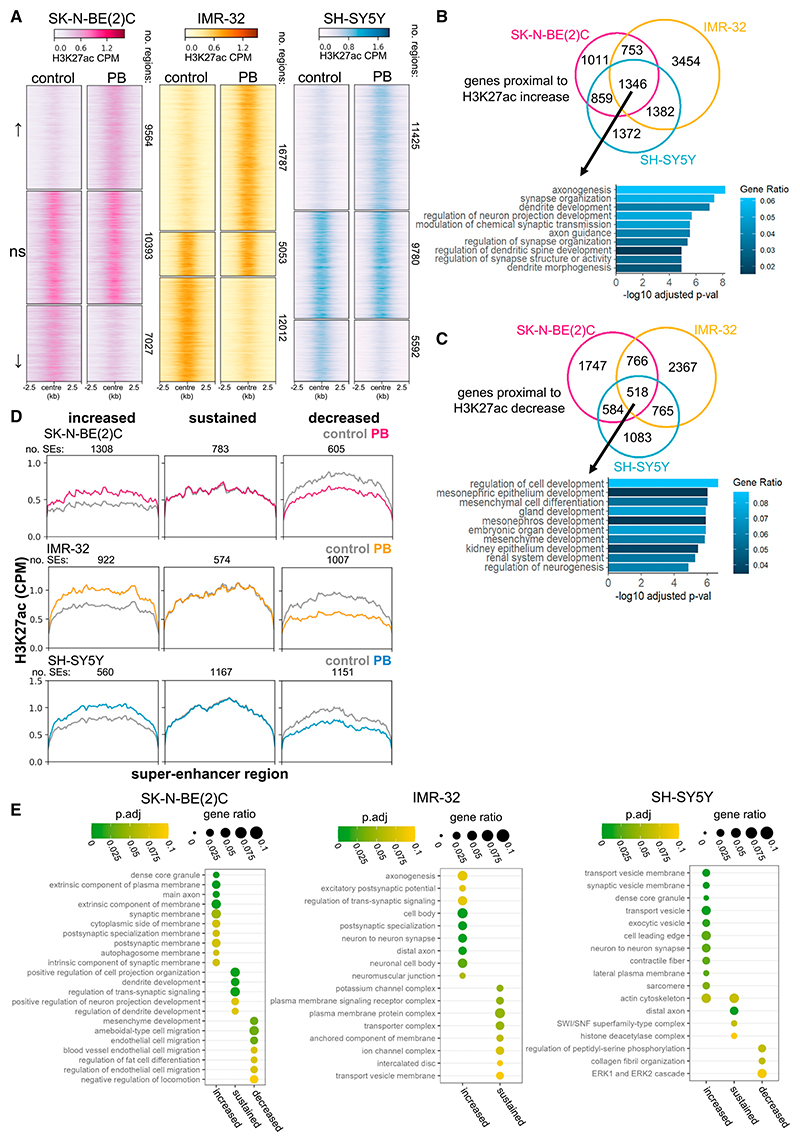
Palbociclib rewires the epigenetic landscape to support differentiation (A) Heatmaps show the CPM (counts per million)-normalized H3K27ac signal (average of five biological replicates) ±2.5 kb around the center of H3K27ac broad-peak loci. Differential H3K27ac marks between PB and control were determined using DiffBind and are grouped as increased (log_2_FC > 0.5 and p.adj < 0.05), no significant change (p.adj > 0.5), and decreased (log_2_FC < −0.5, p.adj < 0.05). Data shown for three cell lines: SK-N-BE(2)C in pink, IMR-32 in orange; and SH-SY5Y in blue. The numbers of increased, unchanged, and decreased H3K27ac marks in each cell line are indicated at the side of each heatmap. (B) Venn diagram shows the crossover of genes proximal (assigned using ChIPseeker) to H3K27ac marks that increase with PB treatment across cell lines; area is proportional to group size. The 10 most significant biological process gene ontology terms associated with the overlapping 1,346 genes are shown. (C) Venn diagram shows the crossover of genes proximal (assigned using ChIPseeker) to H3K27ac marks that decrease with PB treatment across cell lines; area is proportional to group size. The 10 most significant biological process gene ontology terms associated with the overlapping 518 genes are shown. (D) Profiles of average normalized H3K27ac coverage (control in gray, PB in color) across scaled super-enhancer regions. The super-enhancers are grouped for each cell line by how the total H3K27ac signal in the region changes with PB treatment: increased, sustained, or decreased. The number of super-enhancers in each group is indicated. (E) Gene ontology analysis of genes associated with super-enhancers that increase, maintain, or decrease in H3K27ac signal after PB treatment in SK-N-BE(2)C, IMR-32, and SH-SY5Y cells. The top ten terms for each group are shown; there were no significant terms for IMR-32 decreased SEs. See also Figure S3.

Given the strong epigenetic component known to drive neuroblastoma tumors,^15,47,48^ we investigated the impact of PB treatment on the epigenetic land-scape of neuroblastoma cells. We conducted H3K27ac chromatin immunoprecipitation sequencing (ChIP-seq) (a marker of active enhancers^49^) in SK-N-BE(2)C, IMR-32, and SH-SY5Y cell lines after longer PB treatment (5–7 days) (Figure S3A), when extensive morphological differentiation was observed. DiffBind^50^ was used to determine regions with significantly altered deposition of H3K27ac. This analysis identified extensive changes in H3K27ac-marked regions following PB treatment in all three cell lines (Figure 3A). Common to at least 2 of 3 cell lines are 5,641 increased sites of H3K27ac deposition, 2,561 decreased sites, and 2,830 sites with sustained H3K27ac deposition before and after treatment. Differential H3K27ac marks were more likely to be distal from promoter regions and potentially associated with enhancer regions (Figure S3B). To understand how these epigenetic changes may link to gene expression, the most proximal gene (max distance 100 kb) to the H3K27ac broad peak was assigned as a putative regulatory target. Gene ontology analysis showed that PB treatment resulted in a consistent increase in H3K27ac deposition proximal to genes associated with neuronal development biological processes (Figure 3B), consistent with the changes observed by RNA-seq (Figure 2). Genes proximal to reduced H3K27ac marks are associated with alternative developmental routes including gland, kidney epithelium, and mesenchyme development (Figure 3C).

Changes in cell identity are governed by critical lineage-defining genes that are typically regulated by clusters of enhancers with high H3K27ac signal,^15^ termed SEs. To interrogate the effect of PB treatment on SE regions, H3K27ac broad peaks were stitched into clusters and a consistent normalized H3K27ac signal threshold used to identify SEs, enabling comparisons between cell lines and conditions (Figure S3C). SEs were grouped as either increased, sustained, or decreased, based on the change in total H3K27ac signal in the SE between the PB and control condition in each cell line (Figure 3D); specific examples of SE regions with a consistently increased, maintained, or decreased H3K27ac signal across the three cell lines are shown in Figure S3D. Gene ontology analysis showed that genes within 100 kb of SEs that have increased H3K27ac deposition upon PB treatment are related to neuron-to-neuron synapses, axons, dense-core granules, and in the case of SH-SY5Y cells, contractile fibers (Figure 3E). Interestingly, sustained SEs that are present before and after PB treatment are linked to genes associated with plasma membrane complexes, ion channel complexes, and dendrite development (Figure 3E), perhaps reflecting the state of ADRN-type neuroblastoma cells that may already be partially primed to differentiate. Together, these H3K27ac broad-peak and SE analyses demonstrate that PB treatment restructures the epigenetic landscape of neuroblastoma cells to favor neuronal differentiation and axonogenesis as well as restrict alternative developmental pathways.

### PB inhibits tumor growth in *in vivo* mouse models of neuroblastoma

**Figure 4:**
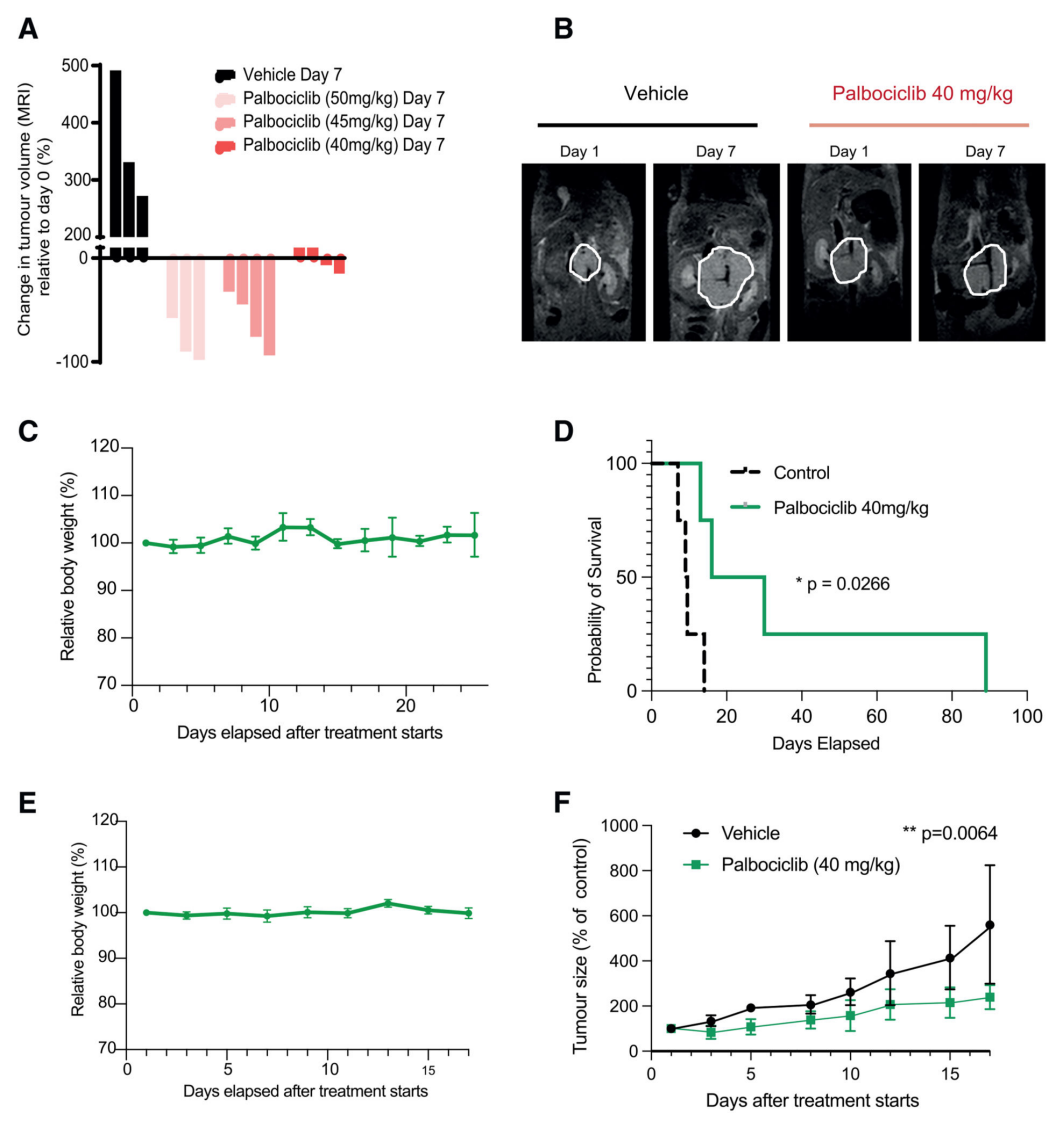
Palbociclib inhibits tumor growth in *in vivo* mouse models of neuroblastoma (A) Waterfall plot documenting relative changes in tumor volume at day 7 in the Th-*MYCN* GEM model with vehicle or palbociclib at the indicated doses. Each line on the graph represents one mouse. (B) Representative MRI sections of mice at days 1 and 7 of treatment with vehicle or palbociclib (40 mg/kg). The white lines indicate the tumor circumference. (C) Graph of relative body weight vs. days elapsed after palbociclib treatment starts (n = 4 mice). (D) Kaplan-Meier survival curve of mice treated with palbociclib versus control untreated mice (n = 4 mice for each condition). Log-rank (Mantel-Cox) test, p = 0.0266. (E) Graph of tumor size percentage relative to day 0 vs. days after treatment starts (n = 6 mice for each condition, control vehicle, or PB treatment [40 mg/ kg]). Mixed two-way ANOVA, p = 0.0064. (F) Graph of relative body weight vs. days elapsed after palbociclib treatment starts (n = 6 mice).

We surmised that the dual anti-proliferative and pro-differentiation effects of PB we observed *in vitro* could provide a therapeutic opportunity. To investigate this insight in a more clinically relevant *in vivo* setting, we used the extensively characterized Th-*MYCN* genetically engineered mouse (GEM), known to mirror many clinical features of high-risk *MYCN*-driven neuroblastoma,^51,52^ in which MYCN is expressed under the control of the tyrosine hydroxylase (Th) promoter. We optimized the dosage of PB to stabilize tumor growth; MRI scans and tumor growth measurements show 40 mg/kg PB is sufficient to achieve this (Figures 4A and 4B), and monitoring mice weights shows this dosage is well tolerated (Figure 4C). Importantly, we found that PB treatment at this dosage has a significant survival benefit (Figure 4D). To strengthen our *in vivo* findings, we next tested PB in immunocompromised mice xenografted with human neuroblastoma cells IMR-32. PB treatment was found to also effectively restrain tumor growth in this model (Figures 4E and 4F). Together, this demonstrates that PB is able to restrain neuroblastoma growth *in vivo* and is an effective and tolerable cytostatic agent.

### PB and RA additively inhibit proliferation of neuroblastoma cells

**Figure 5:**
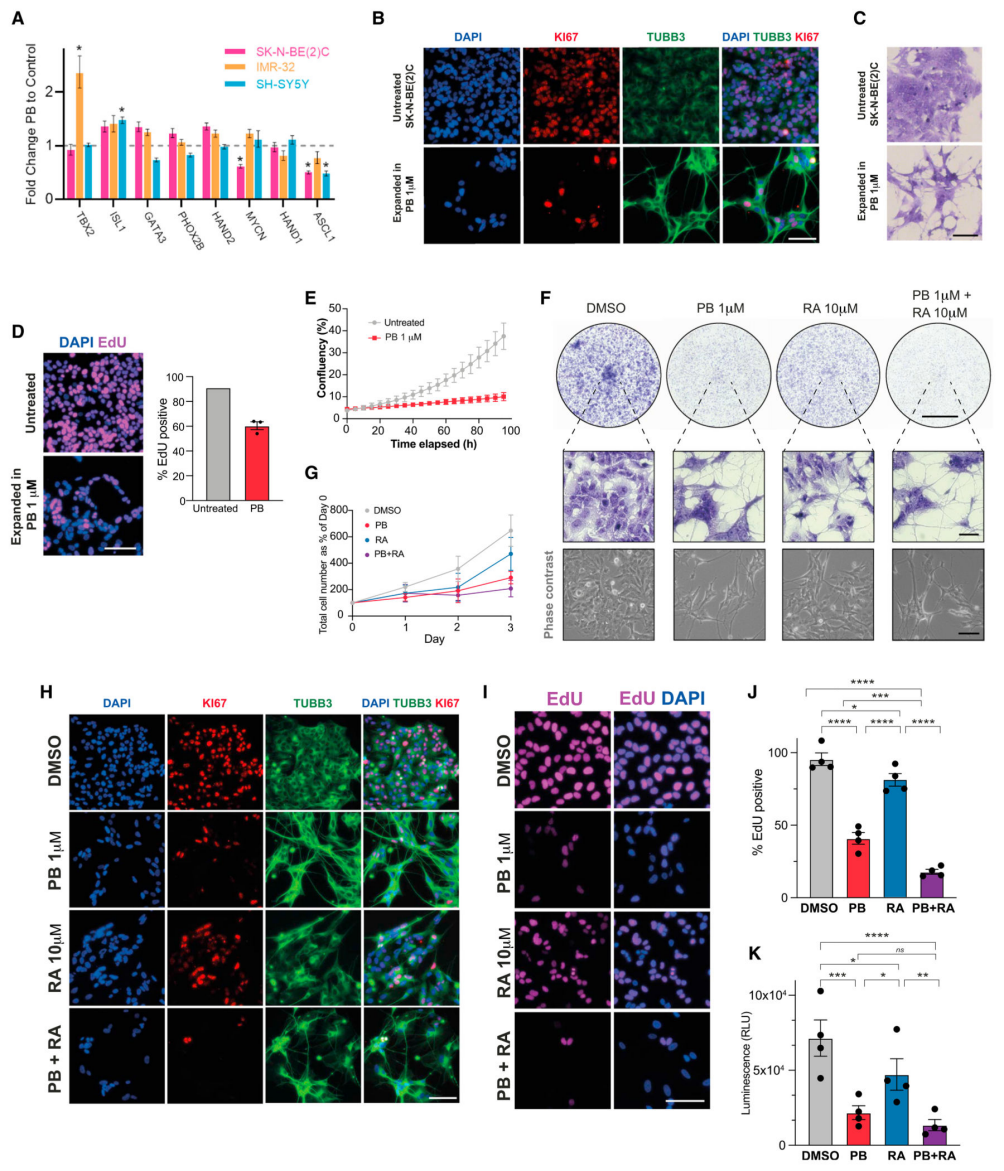
Palbociclib and retinoic acid additively inhibit proliferation of neuroblastoma cells (A) CRC gene expression in RNA-seq data in three neuroblastoma cell lines (n = 5 biological replicates). Data shown as fold enrichment of 5/7 days PB treatment compared with control. Error bars reflect transformed log_2_-fold change (log2FC) standard error values from DESeq2 output. * indicates p.adj < 0.05 and log2FC > 0.5 or < −0.5 in DESeq2 output. (B) Immunocytochemistry analysis of Ki67 (red) and TUBB3 (green) expression in untreated SK-N-BE(2)C cells and cells maintained in 1 μM PB. Representative of n = 3 biological replicates. Scale bars: 100 μm. (C) Crystal violet staining to visualize morphology of untreated SK-N-BE(2)C cells and cells maintained in 1 μM PB. Scale bars: 100 μm. (D) Left: representative fluorescent images of EdU incorporation following a 24-h pulse in untreated SK-N-BE(2)C cells and cells maintained in 1 μM PB. Scale bars: 100 μm. Right: analysis of % EdU-positive cells. Mean ± SEM. n = 3 for PB, where three independent “cultures expanded in PB” were compared with the untreated control. (E) Confluence analysis of untreated SK-N-BE(2)C cells and cells maintained in 1 μM PB. Mean ± SD. For untreated control, n = 6 technical replicates. For PB, n = 3 where three independent “cultures expanded in PB” were compared with the untreated control (each with n = 6 technical replicates). (F) Upper: crystal violet staining of SK-N-BE(2)C cells treated with DMSO vehicle control, PB, RA, or PB + RA for 5 days. Representative of n = 4 biological replicates. Scale bars: 500 μm. Lower: representative phase-contrast and crystal violet staining images of SK-N-BE(2)C cells treated with DMSO vehicle control, PB, RA, or PB + RA for 5 days. Scale bars: 100 μm. Data related to Figure 1E. (G) Daily cell counts of SK-N-BE(2)C cells treated with DMSO vehicle control, PB, RA, or PB + RA for 3 days. Total cell number shown as a percentage of cell number at day 0 for each condition (n = 4 biological replicates). (H) Immunocytochemistry analysis of Ki67 (red) and TUBB3 (green) expression in SK-N-BE(2)C cells treated with DMSO vehicle control, PB, RA, or PB + RA for 5 days. Representative of n = 4 biological replicates. Scale bars: 100 μm. (I) Representative fluorescent images of EdU incorporation following a 24-h pulse in SK-N-BE(2)C cells treated with DMSO vehicle control, PB, RA, or PB + RA for 5 days (pulse began on day 4). Scale bars: 100 μm. (J) Quantification of % EdU-positive cells following 24-h pulse in SK-N-BE(2)C cells treated with DMSO vehicle control, PB, RA, or PB + RA for 5 days. n = 4 biological replicates, mean ± SEM. *p ≤ 0.05; **p ≤ 0.01, repeated measures one-way ANOVA with Tukey’s multiple comparison test. Data related to Figure 1B. (K) Quantification of luminescence (RLU, relative light unit) from CellTiter-Glo cell viability assay. SK-N-BE(2)C cells treated with DMSO vehicle control, PB, RA, or PB + RA for 5 days. n = 4 biological replicates, mean ± SEM. *p ≤ 0.05; **p ≤ 0.01, repeated measures one-way ANOVA with Tukey’s multiple comparison test. See also Figure S4 and Videos S4, S5, and S6.

RA is already used clinically as a differentiating agent in maintenance therapy. Neuronal differentiation of neuroblastoma cells is likely to require both lengthening or arresting the cell cycle in G1 and resetting of the oncogenic CRC.^20,23,53–55^ Indeed, previous evidence shows that RA can reset the ADRN CRC network in neuroblastoma cells and promotes elements of differentiation.^19–21^ Our RNA-seq data identified that PB does not consistently impact the expression levels of ADRN CRC transcription factors^47^ (Figure 5A). Similarly, we did not observe robust changes to the oncogenic CRC-associated SE landscape (as determined by H3K27ac ChIP-seq) after PB treatment (Figure S4A). In keeping with this, investigation of extended PB treatment (>17 days) identified that while all three cell lines displayed neuronal features (Figure S4B), cells were nevertheless capable of expansion. As an example, SK-N-BE(2)C cells maintained in PB continued to divide slowly, as indicated by persistent Ki67 expression, despite exhibiting neurite extension (Figures 5B and 5C). Proliferation was decreased compared with untreated cells, as shown by EdU incorporation, with a 30% decrease in positivity, and by confluency analysis, with a doubling of confluency in ~80 h compared with 24 h for untreated cells (Figures 5D and 5E). These data suggest that while PB treatment alone significantly re-engages the differentiation program in neuroblastoma cells, complete cell-cycle arrest is not achieved and therefore a combinatorial treatment may provide further benefit.

We therefore hypothesized that PB and RA together may further enhance the acquisition of a post-mitotic differentiated state. To investigate this, we treated SK-N-BE(2)C cells with PB (1 μM), RA (10 μM), or PB + RA in combination. Crystal violet staining following 5 days of treatment demonstrated that while RA alone induced limited signs of morphological neuronal differentiation, both PB alone and PB + RA in combination induced extensive neurite formation (Figure 5F). Daily cell counts before and during treatment showed that for all treatments, cell numbers increase or remain constant over time, indicating a lack of cell death (Figure 5G). Live-cell imaging also shows a direct change in morphology during PB and PB + RA treatment, with negligible cell death (Video S4). Ki67 immunocytochemistry, EdU incorporation analyses, and CellTiter-Glo cell viability assays all indicated a significant decrease in cycling cells in PB + RA-treated cells compared with PB alone (Figures 5H–5K), with EdU positivity falling from ~40% to ~18%. Similar results were obtained in IMR-32 and SH-SY5Y cells, with decreased proliferation in PB + RA-treated cells compared with PB alone, as assessed by EdU incorporation, and decreased expression of the established proliferative gene *Ki67*, as well as *E2F2* and *E2F8* (Figures S4C–S4H). PB and PB + RA also increased expression of neuronal markers *STMN4* and *STMN2*, in fitting with live-cell imaging showing morphological changes (Figures S4I and S4J; Videos S5 and S6). These results demonstrate PB + RA further inhibits cell cycling, compared with PB alone, indicating a potential benefit in combining these treatments.

### PB + RA facilitate genome-wide changes favoring reduced proliferation and enhanced differentiation

**Figure 6:**
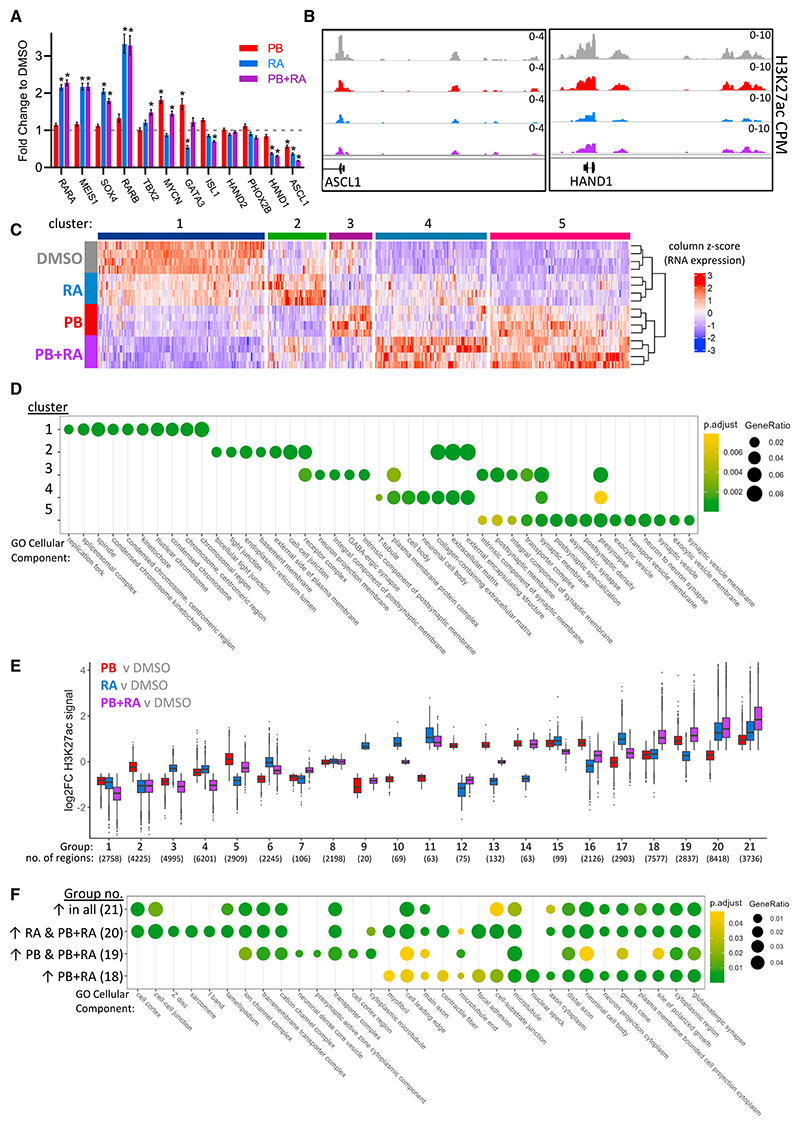
PB + RA facilitate genome-wide changes favoring reduced proliferation and enhanced differentiation (A) CRC gene expression in the RNA-seq data. RARA, RARB, SOX4, and MEIS1 constitute the retino-sympathetic CRC; all other genes are a part of the adrenergic CRC. Data shown as fold enrichment of treatment compared with DMSO control. Error bars reflect transformed log_2_-fold change standard error values from DESeq2 output. * indicates p.adj < 0.05 and log2FC > 0.5 or < −0.5 in DESeq2 output (n = 4 biological replicates). (B) Tracks show normalized H3K27ac signal (average of four biological replicates) at ASCL1 and HAND1 genes after treatment with DMSO, RA, PB, or PB + RA. (C) Heatmap of genes significantly differentially expressed in at least one comparison. A *Z* score has been applied to CPM-normalized RNA-seq counts. Clusters were assigned using k-means clustering on the scaled data. (D) Gene ontology analysis of cellular components for each of the five clusters identified in (C). The top 10 gene ontology terms for each group are shown. (E) Significant differences in H3K27ac marks between conditions were determined using DiffBind and grouped based on how they change with RA, PB, and PB + RA treatment. The boxplots show the log_2_-fold change in H3K27ac for these groups. (F) Gene ontology analysis of cellular components for key upregulated H3K27ac mark groups shown in (D). The most proximal gene (within 100 kb) was assigned to each H3K27ac mark. The top 10 gene ontology terms for each group are shown. See also Figure S5.

To further characterize the response of neuroblastoma cells to PB + RA, RNA-seq analysis and H3K27ac ChIP-seq were conducted in SK-N-BE(2)C cells treated with PB, RA, or PB + RA for 5 days (Figures S5A and S5B). First, we focused on expression of the oncogenic CRC genes. In line with our previous findings (Figures 5A and S4A), we observed that PB alone generally did not significantly change the RNA expression level of oncogenic CRC genes, whereas treatment with RA alone or with PB + RA led to more substantial changes in expression of many CRC-associated factors, including activating expression of the retino-sympathetic CRC genes *RARA, RARB, SOX4*, and *MEIS1* (Figure 6A),^20^ and we saw significant changes in H3K27ac deposition around these genes (Figure S5C). We also observed downregulation in RNA expression and the surrounding H3K27ac marks of the basic-helix-loop-helix (bHLH) transcription factors *ASCL1* and *HAND1* that are usually strongly expressed in proliferating neuroblasts (Figure 6B).^55^ These data suggest that combining PB and RA has a much greater effect on CRC gene expression than PB alone.

Next, we examined the impact of the combination of PB + RA on gene expression more widely. Here, k-means clustering was conducted on genes with significant differential expression in at least one comparison of conditions; this identified five gene clusters in terms of their responsiveness to PB, RA, or PB + RA treatment. Downregulated genes formed one distinct cluster and generally showed a modest downregulation by RA alone, a greater response to PB alone, and an even stronger downregulation with the combined PB + RA treatment (cluster 1, Figure 6C). This cluster of genes was associated with cellular components involved in cell-cycle progression (Figure 6D), consistent with the greater reduction in proliferation we observed with PB + RA (Figures 5F–5K).

Genes upregulated in response to PB and/or RA could be separated into four distinct clusters. Genes that are upregulated only in response to RA are generally associated with cell-cell junctions and the extracellular matrix (cluster 2, Figures 6C and 6D), while genes upregulated only after PB treatment include those associated with postsynaptic membrane components (cluster 3, Figures 6C and 6D). Importantly, we observed a cluster of genes that were more substantially upregulated only after combined PB + RA treatment (cluster 4, Figure 6C), and these genes were associated with the neuronal cell body as well as extracellular matrix cellular components (Figure 6D), consistent with enhanced differentiation. In addition, another subset of genes was upregulated by PB and further upregulated by combined PB + RA treatment (cluster 5, Figure 6C); these were associated with synaptic vesicles, synaptic membranes, and other components associated with neuronal development (Figure 6D).

We also compared how PB and RA, both independently and working together, restructure the epigenetic landscape by using H3K27ac ChIP-seq as a marker of active enhancers. Significantly differential H3K27ac marks between conditions were determined using DiffBind^50^; this was then used to group H3K27ac marks by how they change following PB, RA, and combined PB + RA treatment (Figures 6E and S5B). This identified a range of similar, additive, synergistic, PB-specific, RA-specific, and antagonistic differences in H3K27ac mark regulation (Figure 6E). Of note, for the regions where this mark significantly changed in all three conditions (Figure 6E, groups 1 and 21), the extent of change is greatest in the PB + RA combination, suggesting the drugs have an additive impact on H3K27ac deposition at these sites (Figure 6D). Assessing the location of these H3K27ac marks in relation to transcription start sites showed marks decreased in the presence of PB or PB + RA had a greater tendency to be located proximal to promoter regions, and increased marks were more likely located in more distal regions (Figure S5E).

To understand further the changes in regulation after PB and RA treatment, we focused in on the regions in Figure 6E that have increased H3K27ac signal after PB + RA treatment and assigned the most proximal gene (within 100 kb). Gene ontology analysis showed the regions with increased H3K27ac in all individual conditions, but those that showed the most increase in the combined PB + RA treatment (Figure 6E, group 21) relate to ion channels, synapses, and axons (Figure 6F). In addition to these components, H3K27ac increases that are only found with RA or PB + RA also relate to sarcomere components, while H3K27ac increases that are only found with PB or PB + RA are associated with neuronal dense-core vesicles (Figure 6F). Together, these data highlight that both PB and RA alter the epigenetic landscape surrounding genes associated with neuronal differentiation, and via additive and drug-specific regulation, they may enhance these changes when used in combination.

### Dual PB + RA treatment promotes a transcriptional signature favoring patient survival

**Figure 7:**
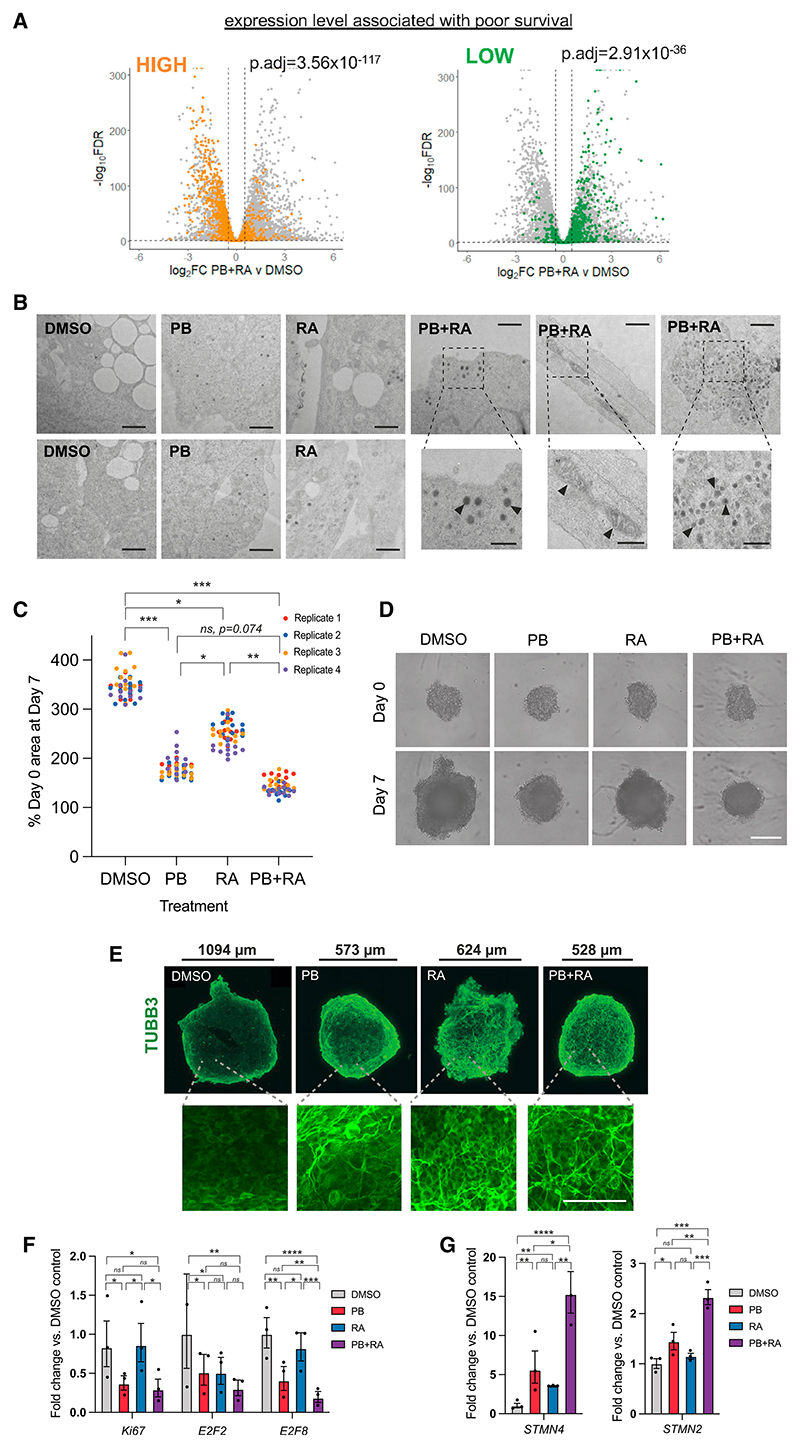
Dual PB + RA treatment promotes a transcriptional signature favoring patient survival and differentiation of tumor spheroids (A) Volcano plots show the change in gene expression in PB + RA compared with the control. Genes with expression levels significantly associated with differential survival in neuroblastoma (determined using the R2 Genomics platform) are highlighted. Genes with high levels being correlated with poor survival are highlighted in orange; genes with low levels being correlated with poor survival are highlighted in green. (B) Transmission electron microscopy images of SK-N-BE(2)C cells treated with DMSO vehicle control, PB, RA, or PB + RA for 5 days (representative of n = 2 biological replicates, scale bars: 1 μm). Higher magnification images for PB + RA condition show dense-core granules of 100–150 nm diameter and mitochondria within neurites, with examples marked by black arrows; scale bars: 500 nm. (C) Percentage SK-N-BE(2)C spheroid area at day 7 of treatment compared with day 0. n = 3 biological replicates, with n = 12 spheroids per replicate, each represented by a single data point. *p ≤ 0.05; **p ≤ 0.01, ***p ≤ 0.001; and ****p ≤ 0.0001, repeated-measures one-way ANOVA with Geisser-Greenhouse correction and Tukey’s multiple comparison test. (D) Representative phase-contrast images of SK-N-BE(2)C spheroids at days 0 and 7 of treatment with DMSO (vehicle), PB, RA, or PB + RA, at the same concentrations used throughout the manuscript. Scale bars: 400 μm. (E) Immunofluorescence images of tumor spheroids stained for TUBB3 (green) at day 7 of treatment. Scale shown for each individual image. Higher magnification images shown with scale bars: 100 μm. (F) qRT-PCR analysis of *Ki67, E2F2*, and *E2F8* expression levels in SK-N-BE(2)C spheroids treated with DMSO vehicle control, PB, RA, or PB + RA for 7 days (~30 spheroids pooled, n = 3 biological replicates). Mean ± 95% confidence interval (CI). *p ≤ 0.05; **p ≤ 0.01, ***p ≤ 0.001; and ****p ≤ 0.0001, repeated measures one-way ANOVA with Tukey’s multiple comparison test. (G) qRT-PCR analysis of *STMN4* and *STMN2* expression levels in SK-N-BE(2)C spheroids treated with DMSO vehicle control, PB, RA, or PB + RA for 7 days (~30 spheroids pooled, n = 3 biological replicates). Mean ± 95% CI. *p ≤ 0.05; **p ≤ 0.01, ***p ≤ 0.001; and ****p ≤ 0.0001, repeated measures one-way ANOVA with Tukey’s multiple comparison test. See also Figure S6.

We next sought to assess how transcriptional changes that occur in response to PB + RA may point to potential benefits of such a treatment to patient survival. Using data from the R2 Genomics platform (http://r2.amc.nl), we identified two gene sets that positively and negatively correlate significantly with poor patient survival in neuroblastoma. We then looked at how these gene sets change in expression after combined PB + RA treatment of SK-N-BE(2)C cells, compared with the vehicle-treated control. Strikingly, genes where high expression is usually associated with poor patient survival were strongly downregulated by PB + RA, while genes where low expression is associated with poor survival were significantly upregulated (Figure 7A). Thus, PB + RA is able to drive a transcriptional program that is likely to be highly beneficial for patients.

### Treatment with PB + RA drives ultrastructural features of mature neuronal differentiation

To determine if the transcriptomic signatures of mature neuronal differentiation observed in cells treated with PB + RA would be reflected as complex structural and functional changes associated with post-mitotic neurons, we then examined ultrastructural changes of treated SK-N-BE(2)C cells by transmission electron microscopy (TEM). This analysis identified striking ultrastructural features that are present after 5 days PB + RA treatment, consistent with hallmarks of neuronal cells, including filaments, microtubules, and mitochondria within neurites, as well as prominent dense-core granules localized at cell extremities (Figure 7B).

### PB + RA additively reduces growth and induces differentiation of tumor spheroids

Finally, to test our results in a more complex, clinically relevant culture system, we investigated the effects of PB and RA on tumor spheroids. Spheroids were cultured in a 96 well format and treated upon reaching 200–400 μm in diameter, a size appropriate for drug screening studies.^56^ We observed that spheroid growth was inhibited by PB and PB + RA treatment for all three ADRN cell lines, compared with the DMSO control (Figures 7C, 7D, and S6A–S6D). Immunostaining analysis of these spheroids shows a change in cell morphology, with neurite extension, and upregulation in expression of the neuronal marker TUBB3 with PB and PB + RA treatment (Figures 7E, S6E, and S6F). Fittingly, quantitative reverse-transcriptase PCR (qRT-PCR) analysis showed a downregulation in proliferative markers *Ki67, E2F2*, and *E2F8* and an upregulation in neuronal differentiation markers *STMN4* and *STMN2* with PB or PB + RA (Figures 7F, 7G, and S6G–S6J). In SK-N-BE(2)C spheroids, RA alone had a limited effect, while PB + RA reduced spheroid size, reduced proliferative marker expression, and increased differentiation marker expression more than PB alone. Interestingly, while PB + RA dramatically reduces proliferation and enhances differentiation compared to RA alone in a consistent manner, enhanced differentiation by PB + RA compared to PB alone was variable across lines. Together, our findings suggest that addition of PB to RA as a combinatorial treatment, or use of PB alone in tumors displaying low RA responsiveness, could provide significant clinical benefit in improving the efficacy of neuroblastoma differentiation therapy.

## Discussion

Neuroblastoma is the most common extracranial solid tumor in infants, arising from stalled neural crest cells. With 5-year survival rates remaining at 40%–50% for children with high-risk disease, and many surviving with life-long health effects from cytotoxic therapies,^12,13^ there is an unmet clinical need for novel, kinder therapies to treat infants with this devastating cancer.

In this study, we investigated the response of neuroblastoma cells to the CDK4/6 inhibitor PB. While CDK inhibitors are traditionally thought to exert their effects primarily through cell-cycle control and have been shown to induce senescence in several other cancer types,^57^ we found that PB is able to not only decrease proliferation but also to induce an extensive program of neuronal differentiation in ADRN neuroblastoma cells (Figures 1, 2, and 3).

While the ability of CDK inhibitors to induce cell-cycle arrest may have been the primary reason they have been so extensively examined as therapeutic agents in cancer, a complementary ability to re-engage a latent differentiation program could also be harnessed to contribute to anti-tumorigenic activity. Lengthening the cell cycle may be the trigger for this phenotypic switch^33^; for example, G1 lengthening is thought to increase the time available for cells to respond to differentiation cues.^34^ Our genome-wide analyses show that PB remodels the epigenetic landscape in a manner that favors the expression of neuronal differentiation genes (Figure 3). *In vivo*, PB significantly inhibits tumor growth in mouse neuroblastoma models (Figure 4). While PB-treated neuroblastoma cells show significantly reduced proliferation and extensive differentiation *in vitro* (Figures 1, 2, and 3), our data show that PB alone does not reset the oncogenic CRC, and cells are capable of continued proliferation with sustained treatment (Figure 5). We therefore hypothesized that additional treatment with RA, previously reported to reset the CRC and thereby promoting ADRN neuroblastoma differentiation,^18,20,21^ may drive cells further down the pathway of post-mitotic differentiation. Combined PB and RA treatment significantly reduced proliferation and, importantly, was also effective at further promoting neuroblastoma differentiation in both MYCN-amplified and non-MYCN-amplified neuroblastoma cell lines (Figures 5 and S4). A mature differentiated state was observed both transcriptionally, by increased expression of neuronal differentiation features (Figures 6C and 6D), and functionally by the increased presence of dense-core neurosecretory granules as visualized by TEM (Figure 7B), accompanied by engagement of a differentiation-inducing retino-sympathetic CRC (Figures 6A and S5C).^20^ Driving this phenotypic and transcriptional switch to differentiation has the potential for significant patient benefit (Figure 7A).

Differentiation of neuroblastoma cells has been proposed as an important and untapped way to improve patient therapy in neuroblastoma.^2^ Previous studies aimed at differentiating neuroblastoma cells *in vitro* have used RA either on its own, with limited phenotypic data characterizing the extent of differentiation, or in combination with serum reduction, a method of inducing G1 arrest in cultured cells.^58^ In patients, retinoid treatments are already approved for use in high-risk neuroblastoma as maintenance therapy for minimal residual disease.^8^ Unexpectedly, we find that treatment with RA alone, without serum reduction, has a more limited effect on differentiation or cell-cycle exit in cultured cells, compared with PB alone (Figures 5F–5K and S4C–S4J); this suggests that PB alone is a far more potent differentiation agent than RA. This is somewhat surprising given that RA at least partially dismantles the oncogenic CRC network, while the CRC landscape remains largely intact after PB treatment (Figures 5A and S4A). On testing PB, RA, and PB + RA in tumor spheroid models, we found the effect of RA, and therefore of enhanced differentiation by PB + RA compared with PB alone, to be variable. This perhaps reflects the variable efficacy of RA observed in patients and suggests that some patients may benefit equally from PB or PB + RA treatments. Alternatively, a longer time point may be required to observe RA efficacy in spheroid models. Importantly, PB or PB + RA dramatically reduced proliferation and enhanced differentiation in a consistent manner, compared with RA alone. Our data suggest that treatment with either PB alone or in combination with RA is likely to provide a more robust and clinically effective differentiation response than RA alone, the strategy currently used.

PB is already approved for front-line treatment of HR-positive and HER2-negative breast cancers in combination with endocrine therapy.^30^ A phase I clinical trial of the structurally similar CDK4/6 inhibitor ribociclib has shown promise in pediatric solid tumors,^59,60^ suggesting that CDK4/6 treatment is tolerable. Our *in vivo* data in neuroblastoma mouse models also suggest that PB is a tolerable and effective agent at cytostatic doses. While adoption of multi-drug treatments is a promising avenue for differentiation therapy, there is a lack of clinical trials investigating RA with synergistic compounds.^8^ Here, we find that treatment of neuroblastoma cells with the FDA-approved drug PB additively drives neuroblastoma differentiation together with the current treatment strategy RA. Like neuroblastomas, other neuroendocrine tumors (NETs) are graded according to their differentiation state^61^ and lack clinical trials, with current standards of care limited to surgery, radiotherapy, and cytotoxic treatments.^62,63^ Promoting differentiation and inhibiting self-renewal simultaneously is an interesting avenue for future exploration in poorly differentiated NETs and indeed other cancer types driven by immature, stem-like cells.^64^ These approaches hold great promise for improving the efficacy of differentiation therapies in a number of tumor types.

### Limitations of the study

The subset of neuroblastoma cell lines used in this study were chosen to cover a mixture of genetic backgrounds. Studying the effect of PB and RA on a greater panel of cell lines would strengthen our conclusion that PB or PB + RA will reactivate the latent differentiation potential of neuroblastoma cells independent of genotype. Our work suggests that enhanced differentiation by PB + RA, compared with PB alone, was variable across cell lines; addition of further cell lines would aid our understanding of whether PB alone is sufficient in tumors displaying low RA responsiveness. Finally, to progress this work toward the clinic, future studies in mouse models of neuroblastoma are needed to characterize the PB + RA combination in an *in vivo* setting, as are treatment of patient-derived tumor samples.

## Star★Methods

### Key Resources Table

**Table:** 

REAGENT or RESOURCE	SOURCE	IDENTIFIER
Antibodies		
TUBB3 (ICC)	Biolegend	801202; RRID: AB_10063408
Ki67 (ICC)	Abcam	ab15580; RRID: AB_443209
Secondary antibody: Goat anti-rabbit IgG (H+L) Highly Cross-Absorbed Alexa Fluor™ 546 (ICC)	ThermoFisher Scientific	A11035; RRID: AB_2534093
Secondary antibody: Goat anti-mouse IgG (H+L) Highly Cross-Absorbed Alexa Fluor™ 488 (ICC)	ThermoFisher Scientific	A11029; RRID: AB_2534088
RB(WB)	Cell Signalling Technology	9309S; RRID: AB_823629
Phospho-RB (WB)	Cell Signalling Technology	9308S; RRID: AB_331472
TBP (WB)	Proteintech	22006-1-AP; RRID: AB_10951514
Secondary antibody: donkey anti-rabbit IRdye800CW (WB)	LI-COR	926-32213; RRID: AB_621848
Secondary antibody: goat anti-mouse IRdye680RD (WB)	LI-COR	926-68070; RRID: AB_10956588
H3K27ac (ChIP-seq)	Abcam	ab4729; RRID: AB_2118291
Chemicals, peptides, and recombinant proteins		
DMEM/F12	Sigma	D8437
FBS	Pan BioTech	P40-37500
Pen/Strep	Sigma	P0781
BSA	Fisher BioReagents™	BP9706-100
Beta-mercaptoethanol	BDH	441433A
Trypsin EDTA 0.25%	ThermoFisher Scientific	25200072
DAPI	Abcam	ab228549
DMSO	Santa Cruz Biotechnology	sc-358801
PowerUp™ SYBR™ Green Master Mix	ThermoFisher Scientific	A25742
Crystal Violet solution	Sigma	V5265
All-trans retinoic acid	Sigma	R2625
Corn oil	Sigma	C8267
Palbociclib (in vitro)	SelleckChem	PD-0332991 HCl, S1116
Palbociclib (in vivo)	MedChemExpress	HY-50767
RIPA buffer	Sigma-Aldrich	R0278
NuPAGE 4-12% BisTris gel	Invitrogen	NP0321BOX
NuPAGE MOPS running buffer	Life Technologies	NP0001
4% Paraformaldehyde in PBS	ThermoFisher Scientific	15670799
Uranyl Acetate	Agar Scientific	R1260A
Epoxy embedding medium Epon812 substitute	Sigma-Aldrich	45345
Glutaraldehyde	Merck	1.04239.0250
Sodium cacodylate	Agar Scientific	R1102
Osmium tetroxide	Agar Scientific	AGR1019
Potassium hexacyanoferrate (II) trihydrate	Sigma Aldrich	P3289
ProLong Gold antifade reagent	ThermoFisher Scientific	P10144
Anti-Adherence Rinsing Solution	STEMCELL Technologies	07010
Critical commercial assays		
RNeasy Mini Kit	QIAGEN	74104
RNase-Free DNase Set	QIAGEN	79254
QuantiTect Reverse Transcription Kit	QIAGEN	205311
Click-iT™ EdU Cell Proliferation Kit for Imaging, Alexa Fluor™ 647 dye	ThermoFisher Scientific	C10340
CellTiter-Glo® Luminescent Cell Viability Assay	ProMega	G7570
BCA protein assay kit	ThermoFisher Scientific	23227
Rapid DNA-seq kit	NEXTFLEX	NOVA-5188-02
Mammalian Genomic DNA Miniprep Kit	GenElute	G1N350
Poly(A) mRNA magnetic isolation module	New England Biolabs	E7490
Ultra II directional RNA kit	New England Biolabs	E7760L
Deposited data		
Raw and processed RNA-seq palbociclib	This manuscript	GSE216273
Raw and processed ChIP-seq palbociclib	This manuscript	GSE216291
Raw and processed RNA-seq palbociclib and retinoic acid	This manuscript	GSE216274
Raw and processed ChIP-seq palbociclib and retinoic acid	This manuscript	GSE236052
Data analysis code	This manuscript	https://zenodo.org/record/8221346 ^65^
Neuroblastoma survival data	R2 Genomics Platform	Tumor Neuroblastoma public – Versteeg - 88 - MAS5.0 - u133p2
Experimental models: cell lines		
SK-N-BE(2)C	Laboratory of Prof. Deborah Tweedle, Newcastle University	N/A
IMR-32	Laboratory of Prof. Deborah Tweedle, Newcastle University	N/A
SH-SY5Y	Laboratory of Prof. John Hardy, UCL	N/A
SH-EP	Laboratory of Prof. Deborah Tweedle, Newcastle University	N/A
GIMEN	CLS Cell Lines Service GmbH, Eppelheim, Germany	AccessionID: CVCL_1232 CLS catalog number 300179
Experimental models: organisms/strains		
*Th-MYCN* mice	William Weiss, UCSF original source. Backcrossed to 129SvJ and bred in house.	Strain: 129SvJ.Cg-Tg(Th- MYCN)41Waw/ICR
NOD *scid* gamma (NSG) mice	JAX original source, bred at Charles River.	Strain: NOD.Cg-Prkdcscid Il2rgtm1Wjl/SzJ Cat #: 05557
Oligonucleotides		
qRT-PCR primers (see Table S1)	Sigma	See Table S1
Software and algorithms		
FIJI/Image J	Open Source	https://imagej.net/software/fiji/
GraphPad Prism version 9.4.1	GraphPad	https://www.graphpad.com/scientific-software/prism/
Image Studio Lite	LI-COR	https://www.licor.com/bio/image-studio-lite/
Incucyte® Base Software	Essen Bioscience	https://www.sartorius.com/en/products/live-cell-imaging-analysis/live-cell-analysis-instruments
Incucyte® Neurotrack Analysis Software Module	Sartorius - Cat. No. 9600-0010	https://www.essenbioscience.com/en/products/peripherals/cell-player-neurotrack-software-module/
Leica LAS X software	Leica	https://www.leica-microsystems.com/products/microscope-software/p/leica-las-x-ls/
NIS-Elements software	Nikon	https://www.microscope.healthcare.nikon.com/en_EU/products/software/nis-elements
cellSens Imaging software	Olympus	https://www.olympus-lifescience.com/en/software/cellsens/
StepOne™ Software	ThermoFisher Scientific	https://www.thermofisher.com/uk/en/home/technical-resources/software-downloads/StepOne-and-StepOnePlus-Real-Time-PCR-System.html
Odyssey infrared imaging system software v3.0.16	LI-COR	https://www.licor.com/bio/support/answer-portal/software/odyssey-application.html
BioRender	BioRender	https://www.biorender.com
MRI software	Horos	https://horosproject.org/
apeglm v1.14.0	Zhu et al.^66^	https://bioconductor.org/packages/release/bioc/html/apeglm.html
bedtools v2.30.0	Quinlan and Hall^67^	https://bedtools.readthedocs.io/en/latest/
bowtie2 v2.4.1	Langmead and Salzberg^68^	https://bowtie-bio.sourceforge.net/bowtie2/index.shtml
ChIPseeker v1.30.3	Yu et al.^69^	https://bioconductor.org/packages/release/bioc/html/ChIPseeker.html
clusterProfiler v4.0.5	Wu et al.^70^	https://bioconductor.org/packages/release/bioc/html/clusterProfiler.html
ComplexHeatmap v2.8.0	Gu^71^	http://www.bioconductor.org/packages/deVel/bioc/html/ComplexHeatmap.html
deepTools v3.5.1	Ramírez et al.^72^	https://deeptools.readthedocs.io/en/deVelop/
DESeq2 v1.32.0	LoVe et al.^45^	https://bioconductor.org/packages/release/bioc/html/DESeq2.html
DiffBind v3.4	Bioconductor	https://bioconductor.org/packages/release/bioc/html/DiffBind.html
dplyr v1.0.10	CRAN	https://cran.r-project.org/web/packages/dplyr/index.html
factoextra v1.0.7	CRAN	https://cran.r-project.org/web/packages/factoextra/index.html
featureCounts v2.0.1	Liao et al.^73^	https://subread.sourceforge.net/featureCounts.html
ggplot2 v3.3.6	CRAN	https://cran.r-project.org/web/packages/ggplot2/index.html
IGV v2.11.0	ThorValdsdóttir et al.^74^	https://software.broadinstitute.org/software/igV/
MACS2 v2.2.7.1	Zhang et al.^75^	https://github.com/macs3-project/MACS/releases/tag/V2.2.7.1
multiQC v1.7	Ewels et al.^76^	https://multiqc.info/
org.Hs.eg.db v3.13.0	Bioconductor	https://bioconductor.org/packages/release/data/annotation/html/org.Hs.eg.db.html
R v4.1.2	CRAN	https://www.r-project.org/
Python v2.7.18	Python	https://www.python.org/download/releases/2.0/
Python v3.9.7	Python	https://www.python.org/download/releases/3.0/
R2Genomics	R2	https://r2.amc.nl
ROSE	Whyte et al.^77^	https://github.com/biomystery/ROSE_young_lab
samtools v1.7	Li et al.^78^	http://www.htslib.org/
snakemake v5.2.1	Molder et al., 2021	https://snakemake.readthedocs.io/en/stable/
STAR v2.6.1a	Dobin et al.^79^	https://github.com/alexdobin/STAR
TrimGalore v0.6.4	Babraham Bioinformatics	https://www.bioinformatics.babraham.ac.uk/projects/trim_galore/
TxDb.Hsapiens.UCSC.hg19.knownGene v3.2.2	Bioconductor	https://bioconductor.org/packages/release/data/annotation/html/TxDb.Hsapiens.UCSC.hg19.knownGene.html
WiggleTools v1.2	Zerbino et al.^80^	https://github.com/Ensembl/WiggleTools

## Resource Availability

### Lead contact

Further information and requests for resources and reagents should be directed to and will be fulfilled by the Lead Contact, Professor Anna Philpott (ap113@cam.ac.uk).

### Materials Availability

This study did not generate new unique reagents.

## Experimental Model And Study Participant Details

### Mice and *in vivo* procedures

All animal experiments were approved by The Institute of Cancer Research Animal Welfare and Ethical Review Body and performed in accordance with the UK Home Office Animals (Scientific Procedures) Act 1986, the UK National Cancer Research Institute guidelines for the welfare of animals in cancer research and the ARRIVE (animal research: reporting in vivo experiments) guidelines.

Th-MYCN mice with the 129/SvJ genetic background were previously described.^52^ Mice were prepared by mating male and female mice at 2-10 months of age. Mice were maintained on a regular diet in a pathogen-free facility on a 12-h light/dark cycle with unlimited access to food and water.

### Cell culture

Neuroblastoma cell lines IMR-32, SH-SY5Y, SK-N-BE(2)C, SH-EP and GIMEN were cultured in DMEM-F12 with L-glutamine (Gibco) supplemented with 10% FBS (Sigma) and 1% Penicillin-Streptomycin (Sigma). Cell lines were verified by submitting genomic DNA for short tandem repeat sequencing and compared with data from the Cellosaurus database. All cell lines were confirmed to be *Mycoplasma* negative and were tested at a minimum of every 3 months. For spheroid formation, cells were seeded in a 96 well format (Sarstedt, 96 well suspension round base, 83.3925.500, washed with STEMCELL Technologies anti-adherence rinsing solution) and centrifuged at 300x g for 3 minutes. Spheroids were treated upon reaching 200-400 μm in diameter, a size appropriate for drug screening studies.^56^

## Method Details

### Drug treatments in cell culture

Palbociclib (PD-0332991 HCl, SelleckChem, S1116) was dissolved in DMSO and used at a final concentration of 1 μM. All-trans retinoic acid (Sigma, R2625) was dissolved in DMSO and used at a final concentration of 10 μM. Media was replaced every 2-3 days.

### Immunocytochemistry

Cells were washed with PBS once before fixation with 4% paraformaldehyde (PFA) for 10 min at room temperature. The PFA was removed, and cells were washed three times for 5 min in PBS to remove excess fixative. Cells were then incubated in PBST (PBS with Triton X-100 0.2%) for 10 min at room temperature for permeabilisation. Cells were incubated in blocking solution (PBS-BSA 3% 0.2% Triton X-100) for 1 hr at room temperature, before overnight incubation at 4 °C with primary antibody in blocking solution (Ki67 1:2000, TUBB3 1:1000). Primary antibody was removed with three 10 min washes in PBST. Cells were then incubated with the appropriate secondary antibody in blocking solution for 1 hr at room temperature. Wash steps were repeated, and cells then incubated in DAPI in PBST (1:10000 dilution) for 15 min at room temperature for nuclear counter-staining then washed once in PBST prior to imaging. Imaging was performed using Leica DMI 6000B Matrix microscope and Leica LAS X software. For 3D cultures, spheroids were stained as above, mounted onto glass slides using ProLong Gold antifade reagent (ThermoFisher) and imaged on Andor Revolution Nikon spinning disk confocal. Images were processed as maximum intensity projections of Z stacks.

### Western immunoblotting

Cells were lysed in RIPA buffer on ice for 20 minutes and centrifuged at 13,000 rpm for 10 minutes. Lysate was retained and quantified using a BCA assay. 30μg protein was run on a 4-12% BisTris gel in MOPS running buffer and transferred to a nitrocellulose membrane. Membranes were blocked and probed in 5% milk-TBS-T. Primary antibodies were incubated at 4°C overnight at 1:1000 dilution and secondary antibodies were used 1:10,000 for 1 hour at room temperature. Images were taken using the Licor Odyssey.

### EdU incorporation assay

For analysis of proliferation rates, cells were incubated in media supplemented with 10 μM EdU for 24 h (beginning at day 4 of five-day drug treatment). Cells were then fixed at day 5 in 4% PFA for 10 min at room temperature and stained with the Click-iT EdU Alexa Fluor 647 assay kit (Life Technologies) according to manufacturer’s instructions. Imaging was performed using Leica DMI 6000B Matrix microscope and Leica LAS X software. The total cell number was determined by DAPI staining. Quantification was performed using the Image thresholding and Particle Analysis functions on FIJI software.

### Confluence analyses

Confluence analysis and growth curves were determined using the Incucyte® live cell imaging system (Essen Bioscience). Cells were plated in triplicate wells of a 24-well plate and imaged periodically until confluence of the control cells was reached. For visualisation of confluency by crystal violet staining, 6 well plates were fixed on Day 5 of treatment in 4% PFA for 10 min at room temperature. Plates were washed with PBS, before incubation with 0.5% aqueous crystal violet staining solution for 30 minutes at room temperature. Plates were washed gently with deionised water and allowed to dry before imaging on an Olympus IX51 microscope with cellSens Dimension imaging software.

### Live-imaging and Neurotrack analyses

Live-imaging was performed using the Incucyte® Live-Cell Analysis System (Sartorius). Cells were plated in duplicate wells of a 24-well plate and imaged ever 6 hours over a period of 5 days of treatment. All videos are representative of n=3 experiments. Videos were stabilised using the image stabiliser plugin for ImageJ and exported at 10 frames per second (K. Li, https://www.cs.cmu.edu/~kangli/code/Image_Stabilizer.html, February, 2008). Automated segmentation of neurite outgrowth and quantification of branch points was performed using the Incucyte Neurotrack Analysis Software Module.

### Quantitative RT-PCR (qRT-PCR)

Cells were lysed using RLT buffer and RNA extracted using the RNeasy Mini kit (Qiagen). RNA concentration was determined using a NanoDrop™ Spectrophotometer. Within each experiment, the same amount of RNA was inputted for cDNA synthesis. Reverse transcription was performed using the QuantiTect Reverse Transcriptase Kit (Qiagen). cDNA was diluted to the required volume using nuclease-free water. Quantitative RT-PCR (qRT- PCR) was performed using gene-specific oligonucleotide primers (Sigma) and SYBR™ Green Master Mix (Thermo Scientific) on an Applied Biosystems StepOne™ Real-Time PCR system. Technical replicates were run to ensure pipetting accuracy and data were analysed using the ddCt method. Replicate Ct values were averaged and normalised to the housekeeping gene, *TBP* (to give dCt). These values were then normalised to a calibrator sample (to give ddCt). Data are presented as Fold change, where this value equals one for the calibrator sample. Data presented as Mean +/- 95% CI, with error bars calculated from ddCt values prior to transformation. Statistics were performed on ddCt values.

### *In vivo* drug treatments

#### *Th*-MYCN *mice*

Transgenic Th-*MYCN* mice were genotyped to detect the presence of the human MYCN transgene. The study was performed using both male and female hemizygous mice, which developed palpable tumors at 45–75 days with 25% penetrance. Mice with palpable tumors and confirmed by magnetic resonance imaging (MRI) were enrolled (day 1) in the study and were treated with 40, 45 or 50mg/kg palbociclib (orally; daily), for 2 weeks. Palbociclib was dissolved in saline. The tumor volume was monitored by MRI at days 1, 3 and 7. Mice were allowed access to sterile food and water ad libitum.

Magnetic resonance images were acquired on a 1 Tesla M3 small animal MRI scanner (Aspect Imaging). Mice were anesthetized using isoflurane delivered via oxygen gas and their core temperature was maintained at 37 °C. Anatomical T2 -weighted coronal images were acquired through the mouse abdomen, from which tumor volumes were determined using segmentation from regions of interest (ROI) drawn on each tumor-containing slice.

#### Xenotransplantation

One million IMR32 neuroblastoma cells were injected subcutaneously into the right flank of NSG mice (female; 6 weeks old) and allowed to establish a murine xenograft model. Mice bearing xenografts with a mean diameter of 5mm were treated with 40 mg/kg palbociclib (orally; daily, dissolved in saline) or vehicle control, for 17 days. Studies were terminated when the mean diameter of the tumor reached 15mm. Tumor volumes were measured by Vernier caliper across two perpendicular diameters, and volumes were calculated according to the formula V=4/3π [(d1+d2)/4]^3^; where d1 and d2 were the 2 perpendicular diameters. The weight of the mice was measured every 2 days and the mice showed no signs of toxicity.

### Transmission Electron Microscopy

SK-N-BE(2)C cells were grown on coverslips and fixed with 4% Paraformaldehyde and 2.5% Glutaraldehyde in sodium cacodylate for 1 hour at RT and then overnight at 4°C. Cells were then washed in dH_2_O 3 x 10 minutes before being post-fixed in 1% osmium tetroxide; 1.5% potassium ferricyanide in dH_2_O for 2 hours at RT before being washed again in dH_2_O for 3 x 10 minutes. Samples were then en-bloc stained overnight at 4°C in 2% Uranyl Acetate (R1260A Agar Scientific powder made up to 2% in water). Cells were washed again 3 x 10 minutes in dH_2_O and then dehydrated in an ethanol series (70%, 95%, 100%) for 10 minutes each and then an additional 2 x 10 minutes at 100% with gentle agitation. The 100% ethanol was exchanged for epoxy embedding medium (Epon812 substitute 45345-250ml-F Sigma-Aldrich) mixed 1:1 with 100% ethanol and left overnight with gentle agitation. 1:1 resin exchanged for 2:1 resin:ethanol for 4-6 hours with gentle agitation and then exchanged for pure epoxy resin. Agar Scientific flat bottom capsules (AGG3547) were filled with epoxy and inverted over the coverslips targeted to areas with cells by eye using the osmium staining. Samples were cured for 48 hours, sectioned at 60nm on a Leica EM UC7 ultramicrotome, and placed onto 200mesh copper grids (Agar Scientific S162 200 Mesh Cu). Grids were post-stained with 1% Uranyl acetate for 6 minutes, washed in 6 filtered dH_2_O droplets, and left to dry, and then stained with Reynolds lead citrate for 2 minutes and washed in 6 filtered dH_2_O droplets. Grids were then imaged on a Hitachi HT7800 Transmission Electron Microscope. 15-20 images were taken at random per condition. Images presented are representative of each condition.

### RNA-sequencing

Cells were cultured in 6 well plates and treated with 1 μM Palbociclib for the indicated duration then subject to RNA extraction with lysis in RLT buffer using the RNeasy Mini kit (Qiagen). DNase digestion was performed using QIAGEN RNase-Free DNase. For Figure 2 control samples were collected at the same time as the 24hr timepoint. RNA samples were polyA selected and libraries made with the NEB Ultra II directional RNA kit. For RNA-seq deposited under GSE216273 five biological replicates were conducted and sequenced on a NovaSeq (Illumina) 100 cycle paired-end. For RNA-seq deposited under GSE216274 four biological replicates were conducted and sequenced on a NovaSeq (Illumina) 50 cycle paired-end.

### H3K27ac ChIP-sequencing

ChIP was performed as in Schmidt et al.^81^ Cells well cultured in 15 cm dishes and treated with 1 μM Palbociclib for the indicated duration. Five biological replicates were conducted. Cells were fixed for 10 minutes with 1% formaldehyde and 0.125 M Glycine (final) subsequently used to quench the fixation. Chromatin was extracted from cells and subject to sonication using a Biorupter for 30 cycles (30 seconds on and 30 seconds off, High) to give DNA fragments of 100-500 bp. 80 ug of chromatin was incorporated into each ChIP and 2 ug of H3K27ac antibody (ab4729) used. DNA fragments retrieved from ChIP were purified using a mammalian genomic DNA miniprep Kit (GenElute). DNA was quantified using a Qubit and libraries prepared using the NEXTFLEX rapid DNA-seq kit Additional H3K27ac ChIP experiments in four biological replicates of SK-N-BE(2)C cells treated with DMSO, 1 μM Palbociclib, 10 μM retinioic acid or 1 μM Palbociclib + 10 μM retinioic acid. For these experiments libraries were prepared with the NEBNet Ultra II DNA library prep kit. Paired-end 100 cycle sequencing was conducted on a NovaSeq (Illumina).

### RNA-seq analysis

Initial paired-end sequencing read processing was conducted by adaptation of an available snakemake pipeline. Reads were trimmed and quality filtered using TrimGalore with a minimum phred score of 20 and then aligned to the hg19 genome using STAR^79^ with quantMode to obtain read counts.

For PB treated SK-N-BE(2)C, IMR-32 and SH-SY5Y samples differential expression analysis was conducted separately for each cell line using DESeq2 with lfcShrink using apeglm for the shrinkage estimator.^45,66^ Differential comparisons made were: 24hPB to control, 5/7dPB to control and 5/7d PB to 24h PB. Significantly expressed genes were classified by an adjusted p-value <0.05 and a log_2_FC > 0.5 or log_2_FC < -0.5. Genes that met these criteria in all three cell lines were used in subsequent k-means clustering analysis. Normalised gene expression (counts per million) in all samples was subject to z-score scaling within each cell line prior to k-means clustering, the resulting clusters were plotted using ComplexHeatmap.^71^ The scaling in the heatmap reflects the z-score for each gene within in each cell line.

For PB and RA treated SK-N-BE(2)C samples differential expression analysis was conducted DESeq2 with lfcShrink using apeglm for the shrinkage estimator.^45,66^ Differential comparisons made were: PB v DMSO, RA v DMSO, PB+RA v DMSO, PB+RA v PB and PB+RA v RA. Significantly expressed genes were classified by an adjusted p-value <0.05 and a log_2_FC > 0.5 or log_2_FC < -0.5. Genes significantly expressed in at least one of these comparisons were retained for clustering analysis. Normalised gene expression (counts per million) in all samples was subject to z-score scaling prior to k-means clustering, the resulting clusters were plotted using ComplexHeatmap.^71^

The list of E2F targets were obtained from the hallmark pathways gene list. For the plotting of specific CRC genes changes (Figures 4A and 6A) the fold enrichment and corresponding error bars reflect the transformed log2FoldChange and lfcSE values from the DESeq2 results respectively. Genes lists regarding expression level association with patient survival were obtained from the R2 Genomics platform (data set: Tumor Neuroblastoma public – Versteeg – 88 – MAS5.0 – u133p2), one list of genes with low expression significantly associated with poor survival in neuroblastoma and another list of genes with high expression significantly associated with poor survival in neuroblastoma. These genes were mapped on to RNA-seq data comparing gene expression between PB+RA treated samples and controls.

### H3K27ac ChIP-seq analysis

Paired-end reads were trimmed and quality filtered using trimGalore or fastp and aligned to hg19 using bowtie2.^68^ Broad peaks were called used MACS2^75^ with input samples as a reference. DiffBind was then used to create a consensus set of peaks present in at least three of five replicates of a given condition and to call differential H3K27ac marks with the parameter summits=FALSE and with the input samples as a reference. Significantly differential H3K27ac marks were assigned by an adjusted p-value <0.05 and a log_2_FC > 0.5 or log_2_FC < -0.5.

For additional H3K27ac ChIP-seq detailed in Figures 6 and S5, DiffBind consensus peaks were defined as those in at least two of four replicates in a given condition. For the groups of regions in Figure 6E, significantly differential H3K27ac signal for RA, PB, or PB+RA compared to the DMSO condition was determined using DiffBind and then these were grouped based on the direction of change across the three conditions. The not significant group was defined by an FDR > 0.5.

Average H3K27ac mark profiles were created using deeptools bamCoverage^72^ to first create a bigwig for each sample with CPM normalisation to account for library size, followed by wiggleTools^80^ to obtain a mean bigwig across all replicates. DeepTools plotHeatmap^72^ was used to show the H3K27ac mark genomic loci, the heatmap scales were capped at the 95^th^ percentile of H3K27ac signal to prevent the impact of extreme outliers on the scaling.

The ChIPseeker R package^69^ was used to determine within which features the H3K27ac marks are located. ChIPseeker was also used to assign the most proximal gene to the H3K27ac marks (at a maximum distance of 100kb). The overlap of genes associated with increased or decreased marks across the cell lines are shown in proportional Venn diagrams.

### Super-enhancer analysis

For each sample H3K27ac broad peaks were stitched in to clusters using ROSE^77^ with the exclusion of -/+ 2.5kb around transcription start sites and a maximum stitching distance of 12.5kb. Then a consensus set of clusters was defined for each cell line using bedtools merge across the concatenated stitched region files. FeatureCounts^73^ was then used to count the paired-end reads overlapping these cluster regions across each sample, and normalised for library size based on the total number of read pairs in the sample. An average H3K27ac signal for each region across the replicates for a given condition was then calculated and ROSE used to provide super-enhancer thresholds estimates. To enable comparability between cell lines and conditions and prevent the impact of outliers on the exact tangent determined threshold, the final assignment of super-enhancers was conducted using a threshold consistent across all cell lines and conditions (this was an average normalised total H3K27ac signal of 60). Next, an altered super-enhancer signal level was defined as a log2FC total SE region signal > 0.15 as increased or < -0.15 as decreased, the remainder were classified as sustained. Metaplots across the super-enhancer scaled regions were plotted using plotProfile from the deepTools package.^72^ Specific example H3K27ac tracks were plotted using integrative genomics viewer^74^ with the normalised and averaged bigwigs. To conduct gene ontology analysis, all genes proximal to the super-enhancers (within 100kb) were assigned to the super-enhancer regions using the ROSE_geneMapper.py script in ROSE.

### Gene ontology analysis

Gene ontology analysis was conducted using enrichGO from the clusterProfiler R package,^70^ key parameters used were: OrgD-b=“org.Hs.eg.db”, pAdjustMethod=“fdr”, minGSSize=20, maxGSSize=500. Gene ontology options cellular component or biological process were used. If there were more than ten significant gene ontology terms, the ten most significant were plotted.

## Quantification And Statistical Analysis

Statistical analyses were performed using Prism software, as noted in the figure legends (*, *P* ≤ 0.05; **, *P* ≤ 0.01, ***, *P* ≤ 0.001; and ****, *P* ≤ 0.0001); standard error of the mean calculated from at least three independent experiments, unless noted in figure legends. Biological replicates were considered as different passage numbers of the same cell line plated in independent experiments. Mean and SEM, SD or 95% CIs (qRT-PCR), and n numbers, are shown in the figure legends. Statistical analysis of differential RNA expression was conducted used DESeq2 and with DiffBind for the differential H3K27ac ChIP marks as described in the method details section. For the data in Figure 7A, the dunn test was used to compare the distributions of genes associated with survival.

## Supplementary Material

Supplemental information can be found online at https://doi.org/10.1016/j.devcel.2023.08.028.

Supplementary

Video S1

Video S2

Video S3

Video S4

Video S5

Video S6

## Data Availability

Sequencing data has been deposited in the Gene Expression Omnibus data repository (GEO) under the SuperSeries GSE216292. H3K27ac ChIP-seq -/+ palbociclib in all three cell lines in the SubSeries GSE216291; RNA-seq -/+ palbociclib in all three cell lines in the SubSeries GSE216273; RNA-seq with palbociclib and retinoic acid in SK-N-BE(2)C cells in the SubSeries GSE216274; H3K27ac ChIP-seq with palbociclib and retinoic acid in SK-N-BE(2)C cells in the SubSeries GSE236052. They are publicly available as of the date of publication. Accession numbers are also listed in the key resources table. Custom code associated with analysis in this manuscript has been deposited on Zenodo.^65^ DOI is listed in the key resources table. Any additional information required to reanalyse the data reported in this paper is available from the lead contact upon request.

## Highlights

- Palbociclib inhibits neuroblastoma cell proliferation and drives differentiation
- The transcriptome and epigenome are reprogrammed favoring neuronal differentiation
- Palbociclib inhibits tumor growth in *in vivo* mouse neuroblastoma models
- PB and RA together drive transcriptional changes associated with improved prognosis

## In brief

Ferguson and Gillen et al. show that the CDK4/6 inhibitor, palbociclib, reactivates a latent ability of neuroblastoma cells to undergo differentiation and it improves survival in mouse neuroblastoma models. Moreover, combined treatment of cells with retinoic acid further enhances differentiation, suggesting that adding palbociclib may improve outcomes to the current treatment for neuroblastoma.
