# Supplementary material for: Palbociclib releases the latent differentiation capacity of neuroblastoma cells
Source: Dev Cell. Author manuscript; Available in PMC 2026 Jan 6. (PMC7618569; doi:10.1016/j.devcel.2023.08.028)
Supplement: Supplementary [file EMS211599-supplement-Supplementary.pdf]

**Developmental Cell, Volume *58***

## Supplemental information

# Palbociclib releases the latent differentiation capacity of neuroblastoma cells

**Kirsty M. Ferguson, Sarah L. Gillen, Lewis Chaytor, Evon Poon, Daniel Marcos, Roshna Lawrence Gomez, Laura M. Woods, Lidiya Mykhaylechko, Louis Elfari, Barbara Martins da Costa, Yann Jamin, Jason S. Carroll, Louis Chesler, Fahad R. Ali, and Anna Philpott**

# Supplementary Figure 1

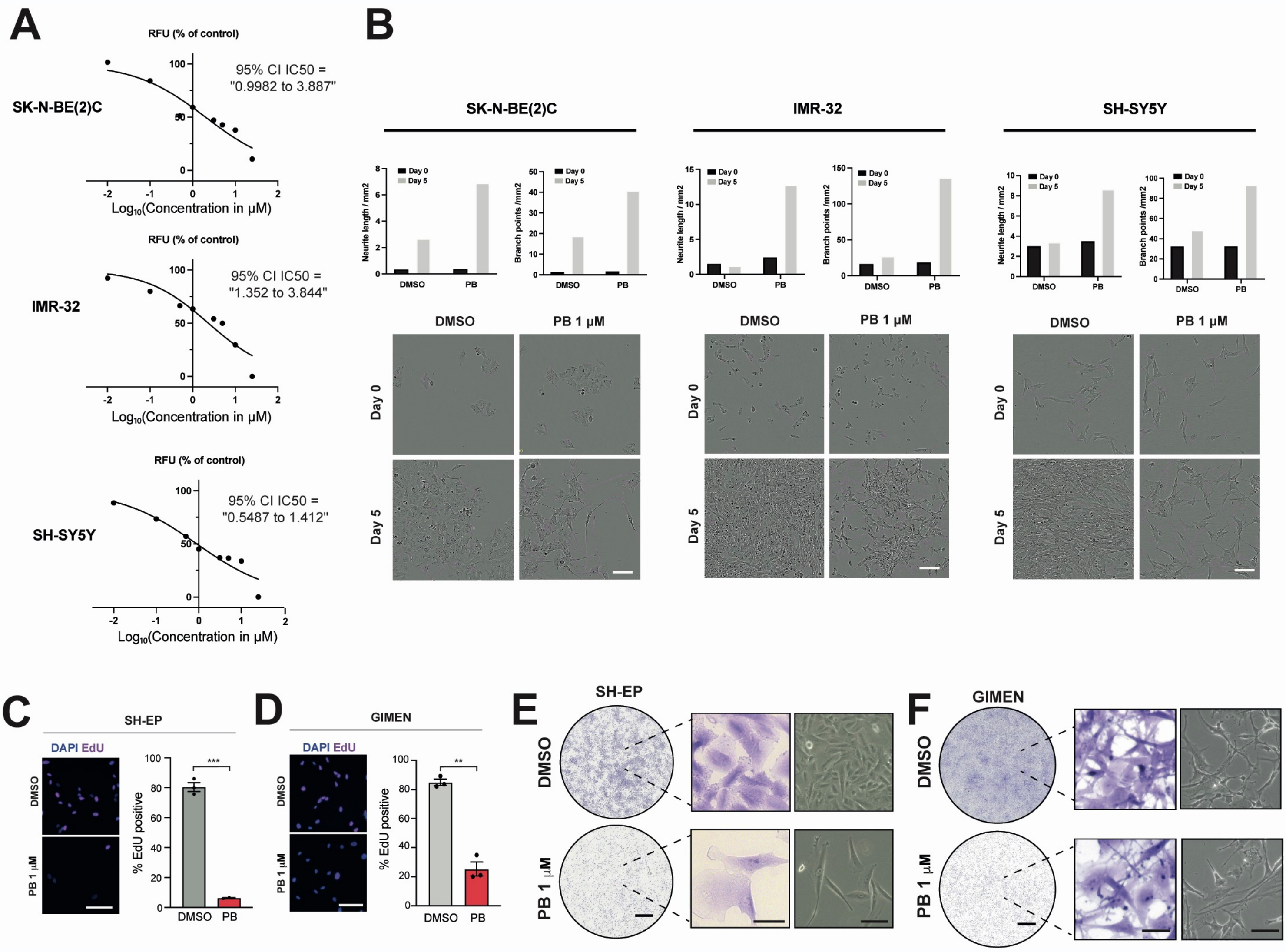


**Supplementary Figure 1 (Related to Figure 1): Palbociclib drives neuronal differentiation in adrenergic neuroblastoma cells.**

(A) IC50 analysis using quantification of luminescence (RLU) from CellTiter-Glo® cell viability assay. SK-N-BE(2)C, IMR-32 and SH-SY5Y treated with a range of PB concentrations from 0 to 25 µM for 5 days. Calculated from n=3 biological replicates, with 4-6 technical replicates per dosage.

(B) Live-cell analysis of neurite length and number of branch points using Incucyte® Neurotrack Analysis Software. For SK-N-BE(2)C, IMR-32 and SH-SY5Y bar graphs show quantification (mean of technical duplicates) at Day 0 (prior to treatment) and Day 5 timepoint for DMSO control and PB (1 µM) treated cells. Phase-contrast images show neurite identification in pink. Scale bar: 100 µm. Representative of n=3 biological replicates.

(C and D) Representative fluorescent images of EdU incorporation following a 24h pulse in SH-EP (C) or GIMEN (D) cells (pulse begun day 4 of 5-day treatment with vehicle (DMSO) or Palbociclib (1 µM)). Scale bar: 100 µm. Analysis of % cells with EdU incorporation. n=3 biological replicates, mean +/- SEM. *, $P \leq 0.05$; **, $P \leq 0.01$, ***, $P \leq 0.001$; and ****, $P \leq 0.0001$ , one-tailed paired t-test.

(E and F) Crystal violet staining of SH-EP (E) or GIMEN (F) cells treated with Palbociclib or DMSO vehicle control for 5 days. Representative of n=3 biological replicates. Right-hand side shows representative phase-contrast images shown prior to fixation and staining. Scale bars: 2 mm, 50 µm and 100 µm.

# Supplementary Figure 2

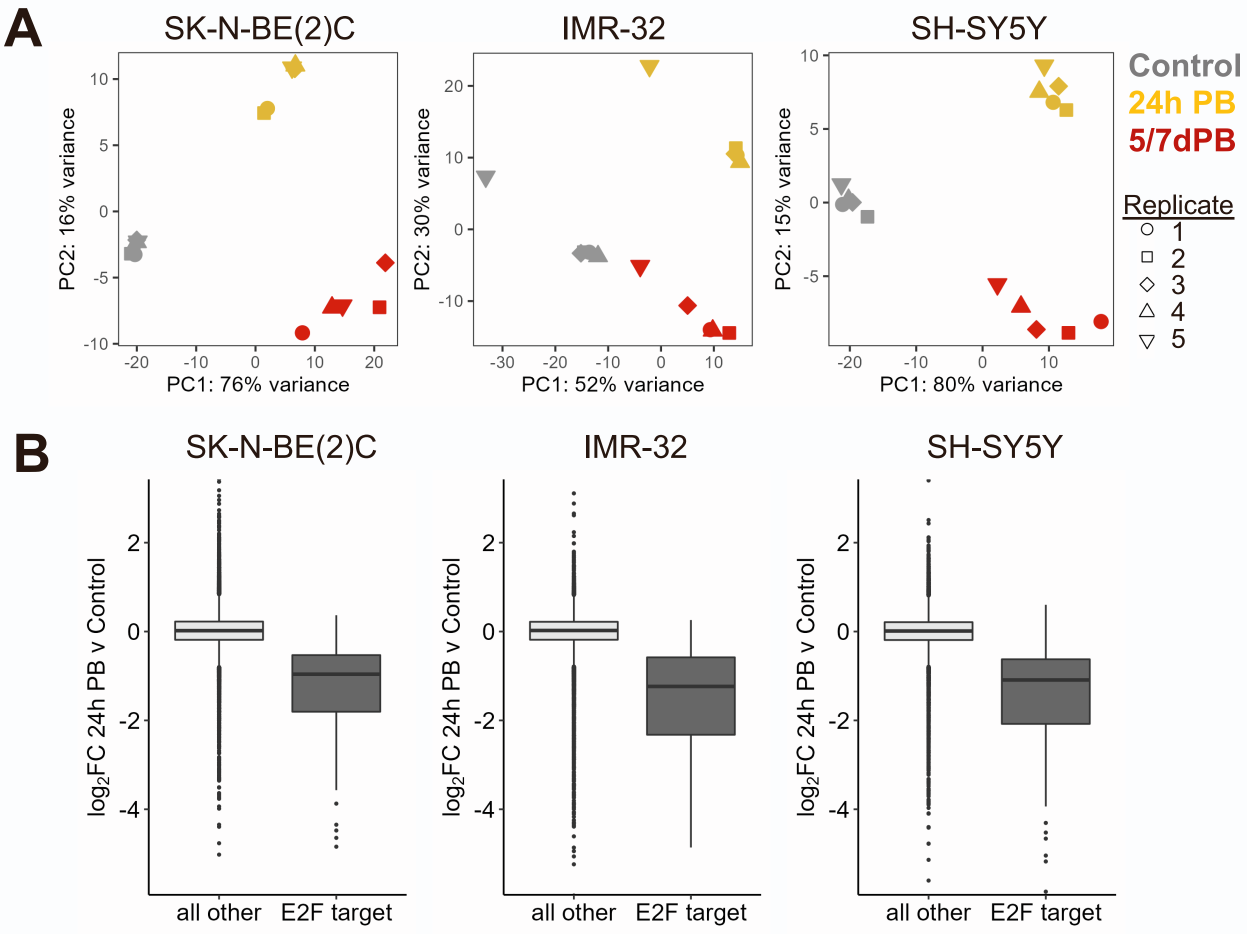


**Supplementary Figure 2 (Related to Figure 2): RNA-seq data quality control.**

(A) PCA plots of the RNA-seq data in each cell line showing the separation of the five biological replicates of control, 24h PB and 5 days PB (IMR-32 and SH-SY5Y) or 7 days PB (SK-N-BE(2)C) treated samples.

(B) Change in expression level of E2F target genes (selected using the hallmark gene ontology set E2F targets) at the early 24h PB time point compared to control.

# Supplementary Figure 3

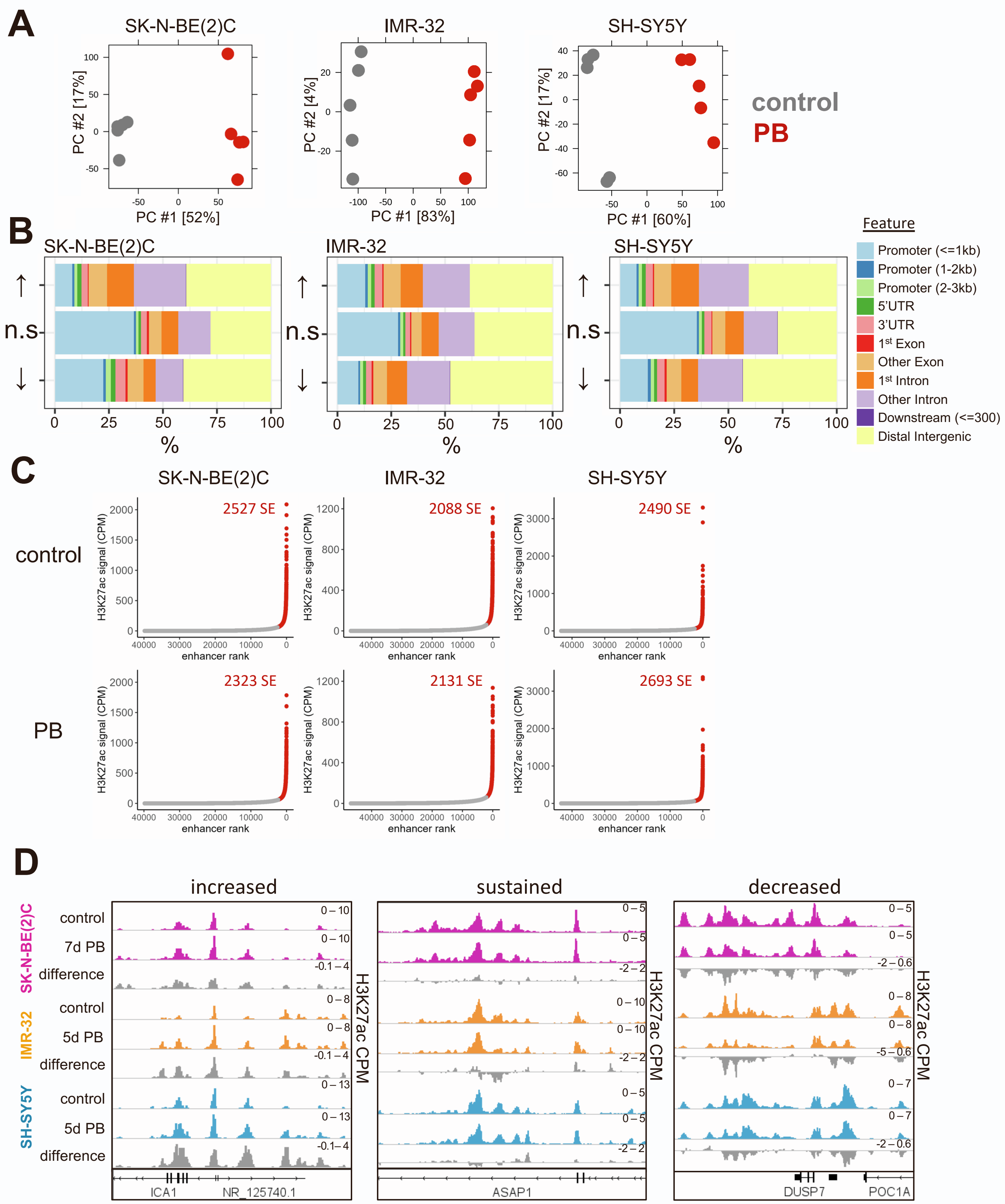

**Supplementary Figure 3 (Related to Figure 3): H3K27ac mark changes following PB treatment in three neuroblastoma cell lines.**

(A) PCA plots of the H3K27ac ChIP-seq data in each cell line showing the separation of the five biological replicates of control and 5d PB (IMR-32 and SH-SY5Y) or 7d PB (SK-N-BE(2)C) treated samples.

(B) Peak annotation for the differential and sustained H3K27ac mark groups (shown in Figure 3A) obtained using ChIPseeker.

(C) Plots show the CPM normalised H3K27ac signal (average of five replicates) v the enhancer rank and the called super-enhancers are highlighted in red. Data is shown for control and PB-treated conditions in each cell line.

(D) Example tracks show the normalised H3K27ac coverage (CPM) for five biological replicates of control and PB treated samples. SK-N-BE(2)C shown in pink, IMR-32 in orange and SH-SY5Y in blue. For clarity the subtracted differential between PB and treated for each cell line is shown in grey. This is shown for: super-enhancer regions with increased, sustained and decreased H3K27ac signal in PB treated cells.

# Supplementary Figure 4

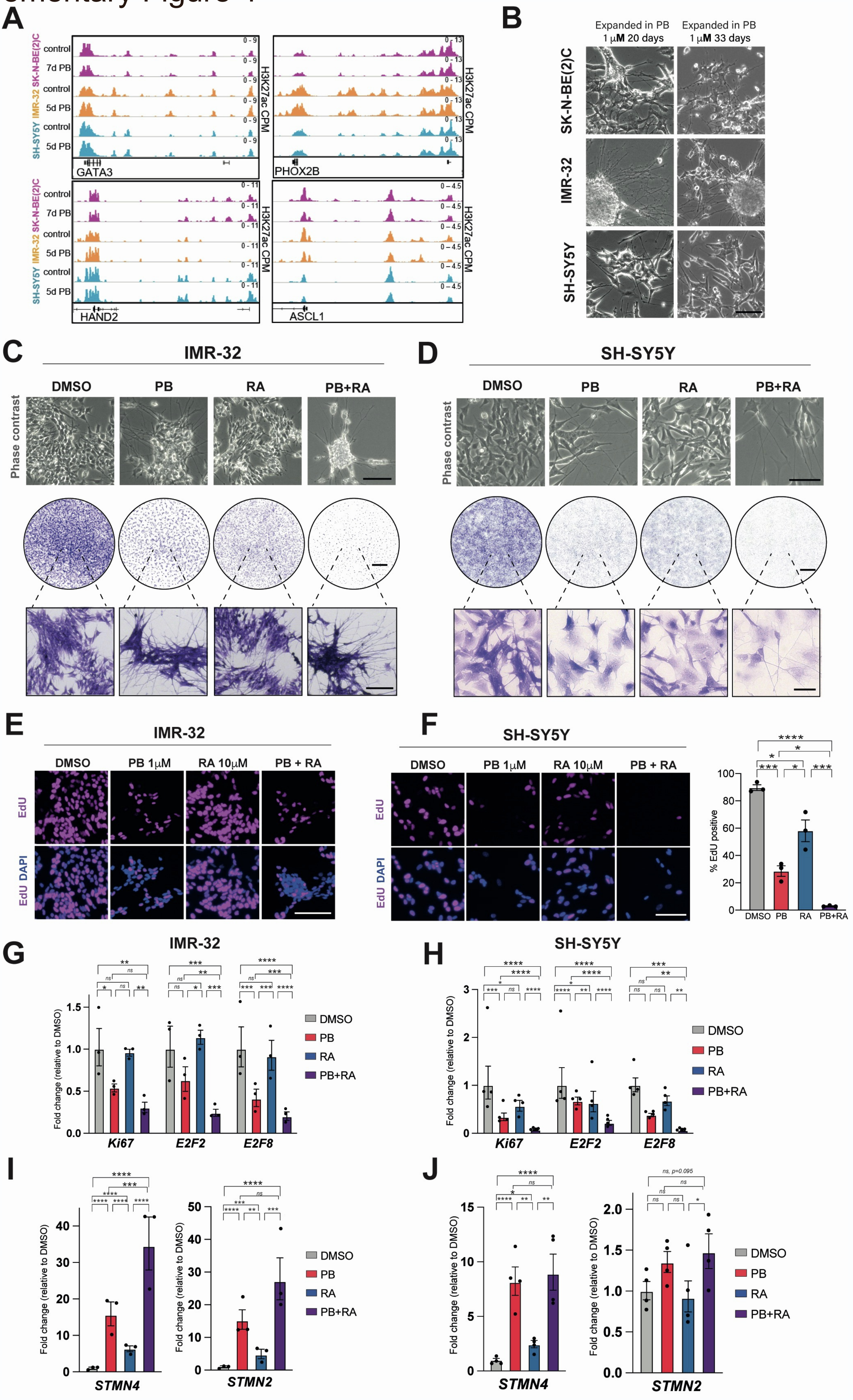

**Supplementary Figure 4 (Related to Figure 5): Palbociclib and retinoic acid additively inhibit proliferation of neuroblastoma cells.**

(A) H3K27ac at the promoters and associated super-enhancer regions of key ADRN CRC genes. Data shown is the average normalised H3K27ac coverage (CPM) for five biological replicates of control and PB treated samples. SK-N-BE(2)C shown in pink, IMR-32 in orange and SH-SY5Y in blue.

(B) Phase-contrast images of SK-N-BE(2)C, IMR-32 and SH-SY5Y cells maintained and passaged in media with 1 µM PB (20 or 33 days). Scale bar: 100 µm.

(C) (Upper) Representative phase-contrast images of IMR-32 cells treated with DMSO vehicle control, PB, RA or PB+RA for 5 days. Scale bar: 100 µm. (Lower) Crystal violet staining of IMR-32 cells treated with DMSO vehicle control, PB, RA or PB+RA for 5 days. Representative of n=3 biological replicates. Scale bars: 2 mm and 100 µm. Note: increased cell clustering and reduced adherence was observed upon PB+RA treatment of IMR-32 cells. Data related to Figure 1E.

(D) (Upper) Representative phase-contrast images of SH-SY5Y cells treated with DMSO vehicle control, PB, RA or PB+RA for 5 days. (Lower) Crystal violet staining of SH-SY5Y cells treated with DMSO vehicle control, PB, RA or PB+RA for 5 days. Representative of n=3 biological replicates. Data related to Figure 1F.

(E) Representative fluorescent images of EdU incorporation following a 24h pulse in IMR-32 cells treated with DMSO vehicle control, PB, RA or PB+RA for 5 days (pulse began on day 4). Scale bar: 100 µm. Increased cell clustering and reduced adherence upon PB+RA treatment of IMR-32 cells made quantification not possible.

(F) (Left) Representative fluorescent images of EdU incorporation following a 24h pulse in SH-SY5Y cells treated with DMSO vehicle control, PB, RA or PB+RA for 5 days (pulse began on day 4). Scale bar: 100 µm. (Right) Quantification of % EdU positive cells. n=3 biological replicates, Mean +/- SEM.. * $P \leq 0.05$; ** $P \leq 0.01$, repeated measures one-way ANOVA with Tukey's multiple comparison test. Data related to Figure 1D.

(G) qRT-PCR analysis of *Ki67, E2F2* and *E2F8* expression levels in IMR-32 cells treated with DMSO vehicle control, PB, RA or PB+RA for 5 days. n=3 biological replicates, Mean +/- 95% CI. *, $P \leq 0.05$; **, $P \leq 0.01$, ***, $P \leq 0.001$; and ****, $P \leq 0.0001$, repeated-measures one-way ANOVA with Tukey's multiple comparison test.

(H) qRT-PCR analysis of *Ki67, E2F2* and *E2F8* expression levels in SH-SY5Y cells treated with DMSO vehicle control, PB, RA or PB+RA for 5 days. n=4 biological replicates, Mean +/- 95% CI. *, $P \leq 0.05$; **, $P \leq 0.01$, ***, $P \leq 0.001$; and ****, $P \leq 0.0001$, repeated-measures one-way ANOVA with Tukey's multiple comparison test.

(I) qRT-PCR analysis of *STMN4* and *STMN2* expression levels in IMR-32 cells treated with DMSO vehicle control, PB, RA or PB+RA for 5 days. n=3 biological replicates, Mean +/- 95% CI. *, $P \leq 0.05$; **, $P \leq 0.01$, ***, $P \leq 0.001$; and ****, $P \leq 0.0001$, repeated-measures one-way ANOVA with Tukey's multiple comparison test.

(J) qRT-PCR analysis of *STMN4* and *STMN2* expression levels in SH-SY5Y cells treated with DMSO vehicle control, PB, RA or PB+RA for 5 days. n=4 biological replicates, Mean +/- 95% CI. *, $P \leq 0.05$; **, $P \leq 0.01$, ***, $P \leq 0.001$; and ****, $P \leq 0.0001$, repeated-measures one-way ANOVA with Tukey's multiple comparison test.

# Supplementary Figure 5

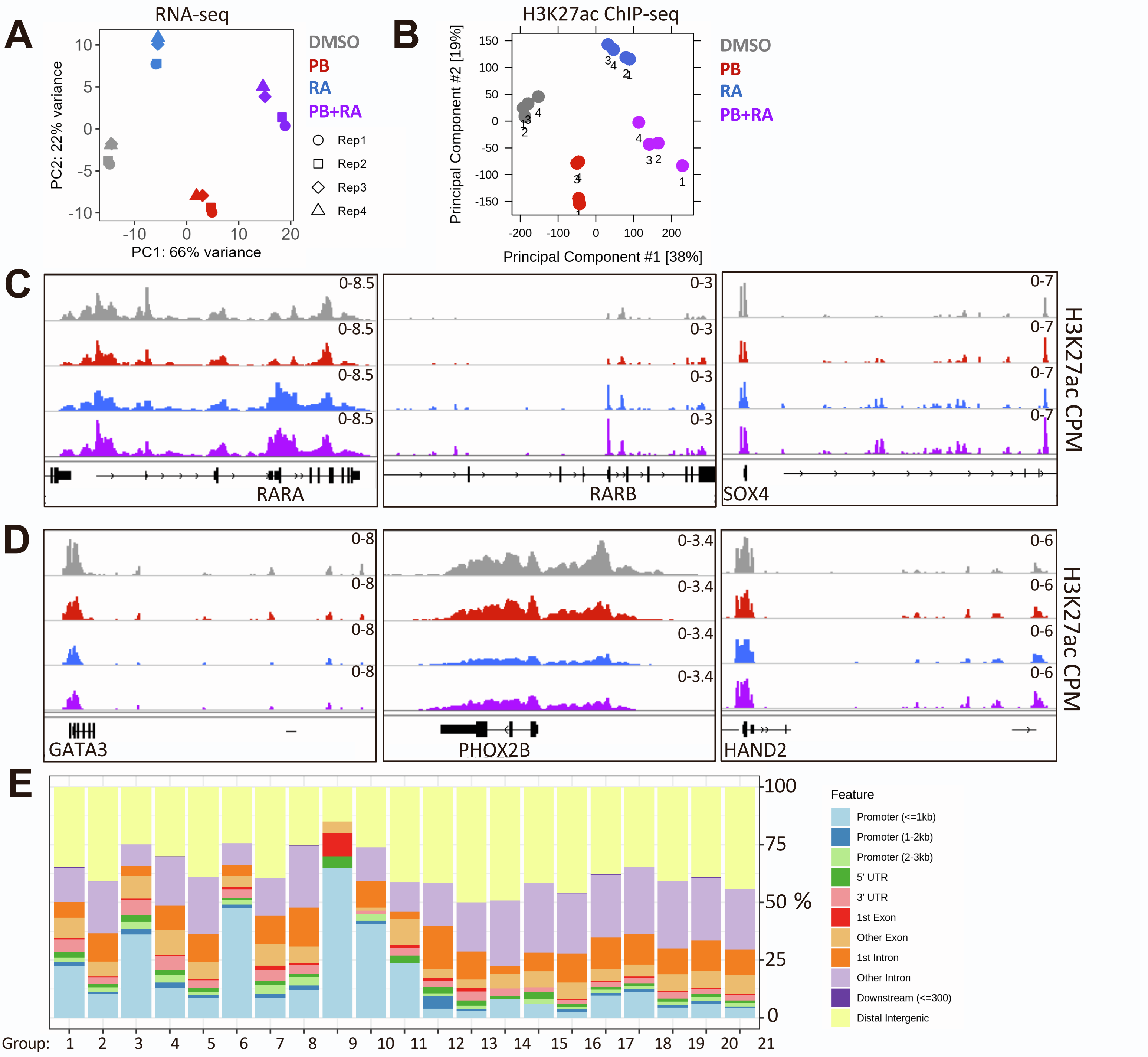


**Supplementary Figure 5 (related to Figure 6): H3K27ac mark changes following PB+RA treatment.**

(A) PCA plots of the RNA-seq data showing the separation of the four biological replicates of DMSO, PB, RA and PB+RA treated samples in SK-N-BE(2)C cells.
(B) PCA plots of the H3K27ac ChIP-seq data showing the separation of the four biological replicates of DMSO, PB, RA and PB+RA treated samples in SK-N-BE(2)C cells.
(C & D) Tracks show normalised H3K27ac signal (average of four biological replicates) genes after treatment with DMSO, RA, PB or PB+RA. (C) at retino-sympathetic CRC genes RARA,RARB and SOX4, (D) at adrenergic CRC components GATA3, PHOX2B and HAND2.
(E) Peak annotation for the H3K27ac mark groups (shown in Figure 6E) obtained using ChIPseeker.

# Supplementary Figure 6

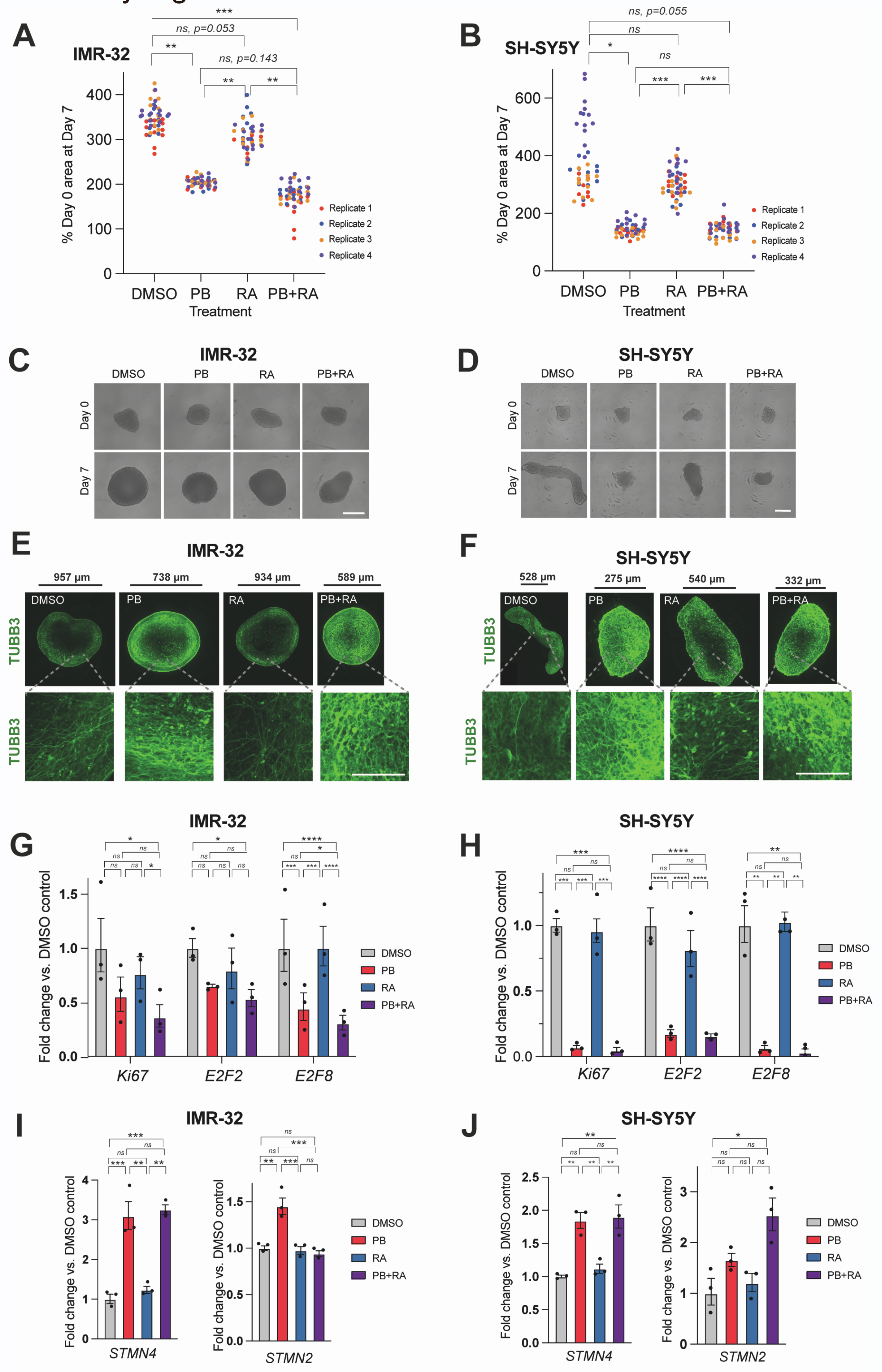

**Supplementary Figure 6 (Related to Figure 7) Dual PB+RA treatment of IMR-32 and SH-SY5Y spheroids.**

(A and B) Percentage IMR-32 (A) or SH-SY5Y (B) spheroid area at Day 7 of treatment compared to Day 0. n=3 biological replicates, with n=12 spheroids per replicate, each represented by a single data point. *, $P \leq 0.05$; **, $P \leq 0.01$, ***, $P \leq 0.001$; and ****, $P \leq 0.0001$, repeated-measures one-way ANOVA with Geisser-Greenhouse correction and Tukey's multiple comparison test.

(C and D) Representative phase-contrast images of IMR-32 (C) and SH-SY5Y (D) spheroids at Day 0 and Day 7 of treatment with DMSO (vehicle), PB, RA or PB+RA, at the same concentrations used throughout the manuscript. Scale bar = 400 µm.

(E and F) Immunofluorescence images of IMR-32 (E) and SH-SY5Y (F) spheroids stained for TUBB3 (green) at Day 7 of treatment. Scale shown for each individual image. Higher magnification images shown with scale bars = 100 µm.

(G and H) qRT-PCR analysis of *Ki67, E2F2* and *E2F8* expression levels in IMR-32 (G) and SH-SY5Y (H) spheroids treated with DMSO vehicle control, PB, RA or PB+RA for 7 days (~30 spheroids pooled, n=3 biological replicates). Mean +/- 95% CI. *, $P \leq 0.05$; **, $P \leq 0.01$, ***, $P \leq 0.001$; and ****, $P \leq 0.0001$, repeated-measures one-way ANOVA with Tukey's multiple comparison test.

(I and J) qRT-PCR analysis of *STMN4* and *STMN2* expression levels in IMR-32 (I) and SH-SY5Y (J) spheroids treated with DMSO vehicle control, PB, RA or PB+RA for 7 days (~30 spheroids pooled, n=3 biological replicates). Mean +/- 95% CI. *, $P \leq 0.05$; **, $P \leq 0.01$, ***, $P \leq 0.001$; and ****, $P \leq 0.0001$, repeated-measures one-way ANOVA with Tukey's multiple comparison test.

**Table S1. Oligonucleotide primer sequences used for qRT-PCR, *Related to STAR Methods.***

| **Oligonucleotide Name** | **Sequence (5' to 3')** |
|---|---|
| Forward primer *Ki67* | GAGGTGTGCAGAAAATCCAAA |
| Reverse primer *Ki67* | CTGTCCCTATGACTTCTGGT |
| Forward primer *E2F2* | CTCTCTGAGCTTCAAGCACCTG |
| Reverse primer *E2F2* | CTTGACGGCAATCACTGTCTGC |
| Forward primer *E2F8* | GAGGCTCAAAGAGGGCAAGCAT |
| Reverse primer *E2F8* | ATGAGCACTGCGTGAGAGGGAT |
| Forward primer *TBP* | GAGCTGTGATGTGAAGTTTCC |
| Reverse primer *TBP* | TCTGGGTTTGATCATTCTGTAG |
| Forward primer *TBP* (gDNA) | CACTCCACTGTATCCCTCCC |
| Reverse primer *TBP* (gDNA) | TGCGGTACAATCCCAGAACT |
| Forward primer *STMN4* | GAAACATGACCCTTGCTG |
| Reverse primer *STMN4* | CTACACTGTCTCCCACAC |
| Forward primer *STMN2* | CCAGAAGAAACTGGAGGCTGCA |
| Reverse primer *STMN2* | GCTTTTCCTCCGCCATCTTGCT |
